# RETREG1‐Mediated Reticulophagy is Essential for Dendritic Cell Maturation and Function in Sepsis

**DOI:** 10.1002/advs.202511021

**Published:** 2026-03-24

**Authors:** Ren‐Qi Yao, Chao Ren, Li‐Yu Zheng, Jing‐Yan Li, Wen‐Feng Wu, Yu‐Xuan Li, Li‐Xue Wang, Yu Duan, Lu Wang, Shuang‐Qing Liu, Peng‐Yi He, Peng‐Yue Zhao, Sen Tong, Zhi‐Xuan Li, Tuo Zhang, Meng‐Yao Wu, Shu‐Ting Wei, Ning Dong, Yao Wu, Hui Zhang, Xiao‐Mei Zhu, Zi‐Cheng Zhang, Guo‐Sheng Wu, Zhao‐Fan Xia, Xiao‐Hui Du, Hong‐Jun Kang, Zui Zou, Dao‐Lin Tang, Yong‐Ming Yao

**Affiliations:** ^1^ Department of General Surgery The First Medical Center of Chinese PLA General Hospital Beijing China; ^2^ Medical Innovation Research Division and the Fourth Medical Center of Chinese PLA General Hospital Beijing China; ^3^ Department of Pulmonary and Critical Care Medicine Beijing Chaoyang Hospital Capital Medical University Beijing China; ^4^ Department of Emergency The Second Hospital of Hebei Medical University Shijiazhuang China; ^5^ Department of Plastic and Reconstructive Surgery Zhongshan Hospital and Shanghai Geriatric Medical Center Fudan University Shanghai China; ^6^ Department of Breast Surgery The First Affiliated Hospital Zhejiang University School of Medicine Hangzhou China; ^7^ Department of Critical Care Medicine The First People's Hospital of Chenzhou Southern Medical University Chenzhou China; ^8^ Department of Critical Care Medicine The First Medical Center of Chinese PLA General Hospital Beijing China; ^9^ Department of Orthopedics The Fourth Medical Center of Chinese PLA General Hospital Beijing China; ^10^ Department of Burn Surgery The First Affiliated Hospital of Naval Medical University Shanghai China; ^11^ Faculty of Anesthesiology Changhai Hospital Naval Medical University Shanghai China; ^12^ School of Anesthesiology Naval Medical University Shanghai China; ^13^ Center For DAMP Biology Department of Surgery UT Southwestern Medical Center Dallas Texas USA

**Keywords:** autophagy, dendritic cells, endoplasmic reticulum, immunosuppression, reticulophagy, sepsis

## Abstract

Dendritic cells (DCs) are crucial antigen‐presenting cells that mediate the interplay between innate and adaptive immunity during lethal infections. Here, we report the key role of reticulophagy regulator 1 (RETREG1), a selective autophagy receptor, in maintaining DC maturation and function in the early stage of sepsis. Mechanistically, activating transcription factor 6 (ATF6) acts as a direct transcription factor regulating RETREG1 expression in response to bacterial lipopolysaccharide‐induced endoplasmic reticulum (ER) stress. RETREG1‐mediated reticulophagy reduces excessive ER stress via the eukaryotic translation initiation factor 2 alpha kinase 3 (EIF2AK3) signaling pathway and inhibits membrane‐associated RING‐CH‐type finger 8 (MARCH8)‐dependent major histocompatibility complex class II (MHC‐II) ubiquitination to maintain antigen presentation in DCs. Consequently, *Cd11c^cre^Retreg1^fl/fl^*, *Retreg1^−/−^*, and *Atf6^−/−^* mice exhibit impaired DC function, leading to immunosuppression and multiple organ failure in experimental sepsis. Exploration of samples from septic patients, combined with single‐cell bioinformatics analysis, further suggests that a deficit in reticulophagy in DCs is associated with the development of human sepsis.

## Graphical abstract

Reticulophagy regulator 1 (RETREG1) maintains dendritic cell (DC) maturation and function in early sepsis. Mechanistically, activating transcription factor 6 (ATF6) acts as a direct transcription factor regulating RETREG1 expression in response to sepsis‐induced endoplasmic reticulum (ER) stress. RETREG1‐mediated reticulophagy reduces excessive ER stress via the eukaryotic translation initiation factor 2 alpha kinase 3 (EIF2AK3) signaling pathway and inhibits membrane‐associated RING‐CH‐type finger 8 (MARCH8)‐dependent major histocompatibility complex class II (MHC‐II) ubiquitination to maintain antigen presentation capacity. Persistent septic challenge impairs this process, compromising DC function and contributing to immunosuppression.

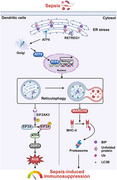

## 1 Introduction

Sepsis remains a leading cause of mortality in intensive care unit (ICU) patients, characterized by multiple organ dysfunction resulting from a dysregulated host response to infection [1]. Data published in 2020 indicate 48.9 million cases and 11 million sepsis‐related deaths worldwide, accounting for 20% of all global deaths [2]. These poor clinical outcomes in septic patients are attributed to aberrant immune responses, including early uncontrolled inflammation followed by persistent immunosuppression [3]. Both the innate and adaptive immune systems exhibit significant dysfunction during septic exposure, with massive loss of effector immune cells and accumulation of immunosuppressive cells [4]. Given the ongoing challenges in effective immune‐monitoring and immune‐modulatory strategies in clinical practice [5], further studies on the mechanisms underlying sepsis‐induced immune dissonance are urgently needed.

Dendritic cells (DCs) are a diverse group of antigen‐presenting cells that originate from bone marrow progenitors [6]. Acting as messengers between the innate and adaptive immune systems, DCs capture and process antigens (foreign substances) and present them on their surface to T cells, thereby initiating an adaptive immune response [7]. A decrease in the number and functional collapse of DCs are associated with the development of sepsis‐induced immune dysfunction and worse prognosis [8, 9]. Our previous studies have demonstrated that DCs are rapidly activated in the early stages of sepsis, but transition to a functionally suppressed state under persistent septic challenge [10, 11]. Animal experiments have also confirmed the benefit of preventing infectious death by enhancing DC function [12]. Therefore, elucidating the underlying mechanisms of the dysregulated response of DCs in sepsis may contribute to developing effective immunomodulatory drugs.

Endoplasmic reticulum (ER) is the largest membrane organelle, responsible for protein translocation, folding, and posttranslational modifications. It also plays an essential role in maintaining functional balance and viability of immune cells [13, 14]. In response to infection, the structure and function of the ER of immune cells undergo dramatic changes, such as accumulation of unfolded and misfolded proteins within the enlarged ER lumen [15]. This ER stress is a pathological cellular feature of sepsis, leading to cell dysfunction and multiple organ failure [16]. We previously found that prolonged ER stress resulted in dysfunction and massive DC loss in experimental sepsis [17, 18], highlighting the potential of targeting ER homeostatic mechanisms to mitigate immune dysfunction in sepsis.

The ER homeostasis can be maintained at multiple levels. One key mechanism is the activation of the unfolded protein response (UPR) through three effector pathways: eukaryotic translation initiation factor 2 alpha kinase 3 (EIF2AK3), endoplasmic reticulum to nucleus signaling 1 (ERN1), and activating transcription factor 6 (ATF6) [15]. These pathways reduce the protein burden in the ER lumen by initiating enzyme‐mediated degradation or limiting mRNA translation [19]. Another critical process is the selective degradation of the damaged ER by autophagy, known as reticulophagy or ER‐phagy [20, 21]. Multiple ER‐localized proteins with at least one microtubule‐associated protein 1 light chain 3 (MAP1LC3/LC3)‐interacting region (LIR) motif have been identified and act as autophagy receptors to recognize damaged ER [21, 22]. Among them, the reticulophagy regulator 1 (RETREG1) is the first identified autophagy receptor that mediates ER clearance via reticulophagy [23, 24]. Although the impaired RETREG1 pathway is implicated in various diseases [25, 26, 27], its modulation and function in sepsis and septic shock remain poorly understood [28, 29].

In this study, we report a previously unrecognized role of RETREG1 in maintaining maturation and function of DCs through reticulophagy during inflammatory stimuli. We demonstrate that ATF6 is a key transcription factor for RETREG1 upregulation under ER stress and confirm that RETREG1‐mediated reticulophagy promotes DC maturation, thereby facilitating an effective adaptive immune response in lethal infections. Our animal and human studies further indicate that activation of RETREG1‐mediated reticulophagy in DCs is a potential strategy to prevent immune dysfunction in sepsis.

## 2 Results

### 2.1 Bacterial LPS Induces Reticulophagy in DCs

To determine the relationship between reticulophagy and activated DCs, we treated immature splenic DCs from mice with lipopolysaccharide (LPS) for 24 and 72 h. Flow cytometry analysis revealed that the expression of CD80, CD86, and major histocompatibility complex class II (MHC‐II) molecules was upregulated after 24 h of LPS stimulation, but returned to baseline levels after 72 h of LPS stimulation (Figure S1A). Enzyme‐linked immunosorbent assay (ELISA) analysis of the effector cytokine, including interleukin (IL)‐12, further confirmed the early impact of LPS on DC maturation (Figure S1B).

To examine the direct effect of LPS‐stimulated DCs on T cell activation, we performed co‐culture experiments using splenic DCs and naive CD4^+^ T cells. DCs stimulated with LPS for 24 h promoted the proliferation and secretion of IL‐2 by CD4^+^ T cells, whereas a 72‐h stimulation did not (Figure S1C,D). Additionally, CD4^+^ T cells co‐cultured with DCs stimulated with LPS for 24 h released higher levels of interferon (IFN)‐γ, with significant reduction observed in IL‐4 production, indicating a polarization toward the helper T cell (Th)‐1 subtype [30] (Figure S1E–G). In contrast, the priming activity of DCs on T cells was inhibited following 72 h of LPS exposure, as demonstrated by reduced cell proliferation and decreased production of IL‐2, IFN‐γ, and IL‐12 (Figure S1B–G). Furthermore, apoptotic activity was augmented in DCs stimulated with LPS for 72 h, when compared to that of 24‐h stimulation, as indicated by the elevated proportion of apoptotic cells and expression level of apoptosis‐related proteins (Figure S2A,B). Nevertheless, administration of zVAD (a Pan‐caspase inhibitor) did not rescue the functional deficit of DCs upon 72 h of LPS stimulation (Figure S2C,D).

**FIGURE 1:**
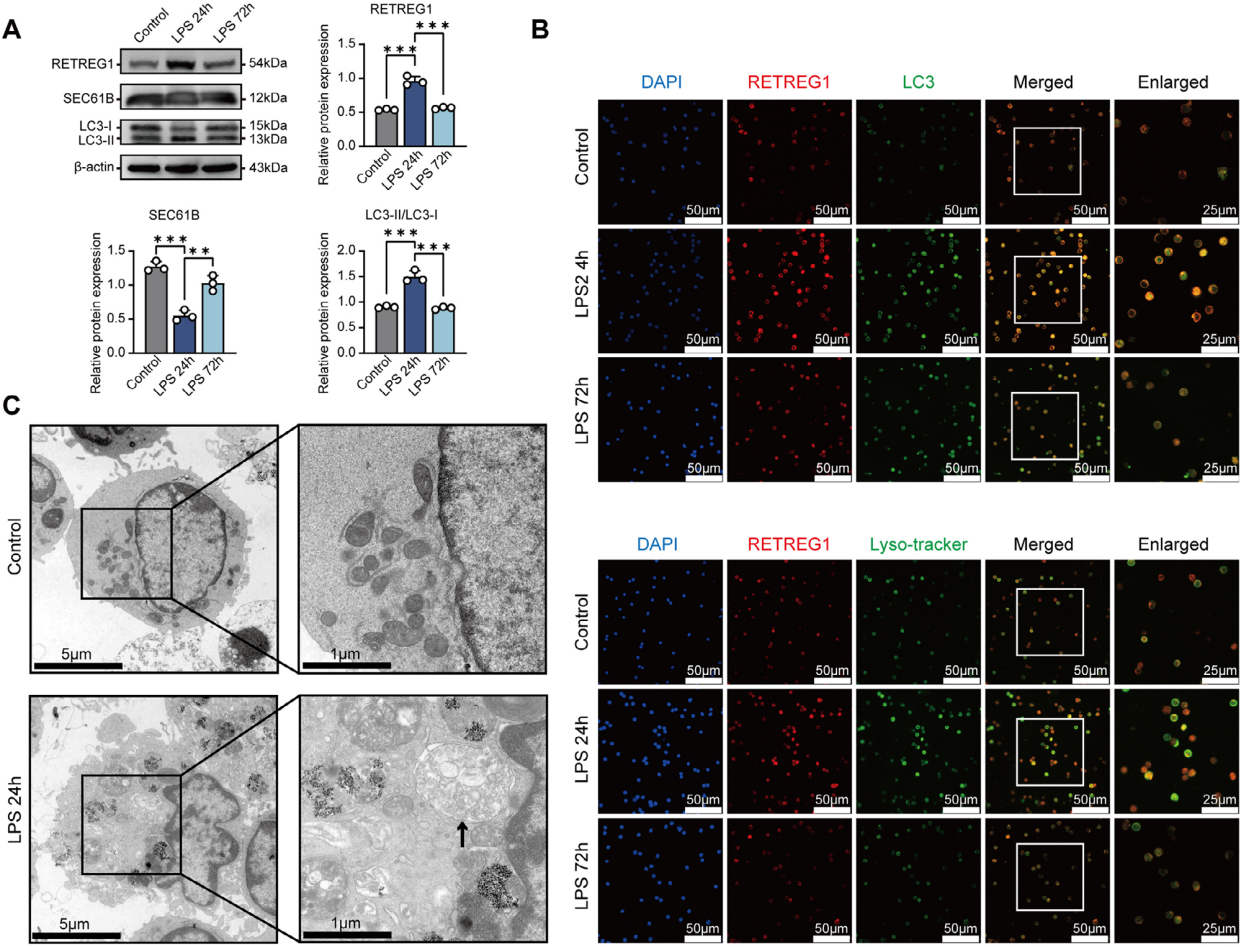
Bacterial LPS induces reticulophagy in DCs. Splenic DCs derived from wild‐type (WT) mice were stimulated with LPS (1 µg/mL) for 0, 24, and 72 h, followed by subsequent experiments. (A) Western blot analysis of the protein expression levels of RETREG1, SEC61B, and LC3 (*n* = 3). (B) Representative confocal immunofluorescence images showing RETREG1 co‐localization with autophagosomes (LC3 puncta, upper panel) or lysosomes (Lyso‐tracker, lower panel). (C) Transmission electron microscopy (TEM) analysis of the formation of ER‐associated autophagosomes (arrows indicating ER within autophagosomes). For panel (A), each protein sample was loaded onto three independent gels, and mean densitometric values were used for inter‐group comparisons. Data in panels (B,C) are representative of three independent experiments. Data in panels (A) represent mean ± SD. Statistical analysis was performed using one‐way ANOVA followed by Tukey's post hoc test. ^**^ *p*< 0.01, ^***^ *p*< 0.001.

We next investigated the time‐dependent effects of LPS on reticulophagy in DCs. Reticulophagy in splenic DCs was evident 24 h after LPS stimulation, characterized by elevated RETREG1 expression, enhanced LC3‐II/LC3‐I conversion, and degradation of the ER‐resident protein SEC61 translocon subunit beta (SEC61B) (Figure 1A). Consistently, immunofluorescence assays revealed co‐localization of RETREG1 with autophagosomes (LC3 puncta) and lysosomes (Lyso‐tracker) in splenic DCs (Figure 1B). Transmission electron microscopy (TEM) showed increased ER‐associated autophagosome formation following 24‐h LPS stimulation (Figure 1C). Conversely, 72‐h LPS exposure suppressed RETREG1 expression and disrupted its colocalization with autophagosomes and lysosomes in DCs (Figure 1A,B). Given that phosphorylation‐dependent ubiquitination of RETREG1 is essential for its reticulophagic activation, we investigated whether LPS similarly induces this posttranslational modification in DCs. As expected, the phosphorylation level of RETREG1 was significantly enhanced after 24 h of LPS stimulation, whereas it returned to the basal level at 72 h (Figure S3A). Concomitantly, LPS treatment induced RETREG1 ubiquitination accompanied by pronounced oligomerization, consistent with recent findings [31, 32, 33] (Figure S3B,C).

Immunoblotting analysis of other known reticulophagy receptors—including cell cycle progression 1 (CCPG1), calcium binding and coiled‐coil domain 1 (CALCOCO1), atlastin GTPase 3 (ATL3), tripartite motif containing 13 (TRIM13), SEC62 homolog, preprotein translocation factor (SEC62), testis expressed 264, ER‐phagy receptor (TEX264), and reticulon 3 (RTN3)—revealed distinct expression patterns following LPS stimulation. Notably, TRIM13, a transmembrane ER‐localized E3 ubiquitin ligase, exhibited marked upregulation at 24 h followed by downregulation at 72 h, paralleling the temporal dynamics of RETREG1 expression (Figure S4A,B). Comparable associations between LPS stimulation and RETREG1‐mediated reticulophagy activation were observed in bone‐marrow‐derived DCs (BMDCs) (Figure S5A–D).

Collectively, these data suggest that RETREG1‐mediated reticulophagic activity is increased by LPS stimulation at 24 h, rather than at 72 h, correlating with the functional activation of DCs.

### 2.2 RETREG1 Mediates Maturation and Activation of DCs in Endotoxemia

To elucidate the potential effects of RETREG1‐mediated reticulophagy on the viability and function of DCs, we isolated splenic DCs from *Retreg1* knockout (*Retreg1^−/−^*) and wild‐type (WT) mice, followed by LPS stimulation for 24 h. Flow cytometry analysis revealed increased apoptotic cell proportions following LPS challenge regardless of *Retreg1* status, with comparable apoptotic rates between LPS‐treated *Retreg1^−/−^* and WT‐DCs (Figure S6A). Consistent with this finding, the levels of cleaved caspase 3 (CASP3) and BCL2‐associated X, apoptosis regulator (BAX), remained unchanged in LPS‐primed DCs lacking *Retreg1*, and BCL2 expression was comparable between the two groups (Figure S6B). These findings indicate that defective reticulophagy is not responsible for LPS‐induced apoptosis in DCs.

**FIGURE 2:**
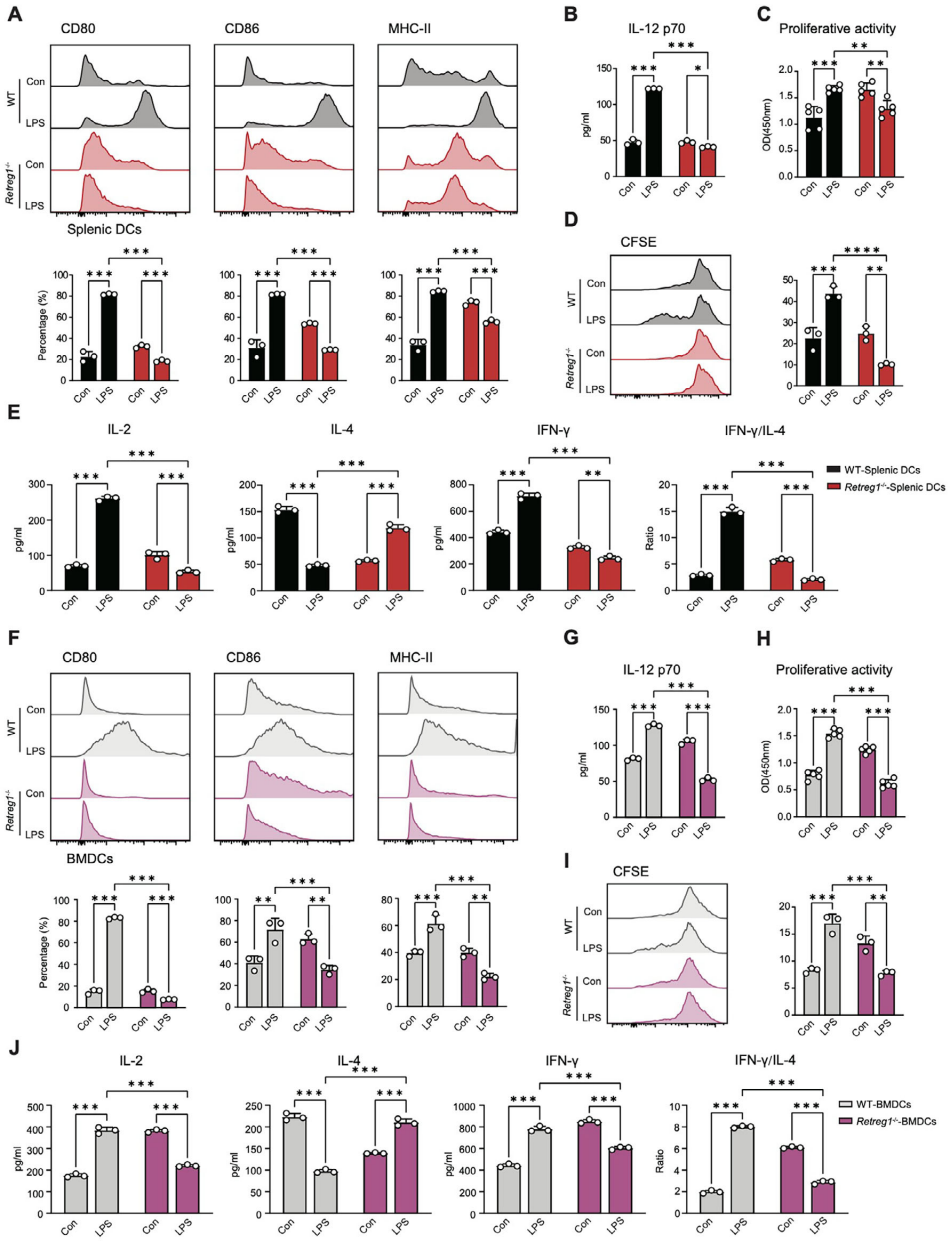
RETREG1 mediates the maturation and activation of DCs in endotoxemia. A‐E Splenic DCs harvested from WT and *Retreg1^−/−^* mice were stimulated with LPS (1 µg/mL) or PBS for 24 h, followed by subsequent assays. (A) Flow cytometry analysis of CD80, CD86, and MHC‐II expression levels (*n* = 3). (B) ELISA analysis of IL‐12 p70 levels in culture supernatants (*n* = 3). (C) CCK8 assay determining the proliferative activity of CD4^+^ T cells co‐cultured with splenic DCs (*n* = 5). (D) Flow cytometry indicating the proportion of divided CD4^+^ T cells stained with CFSE dye (*n* = 3). (E) ELISA analysis of IL‐2, IL‐4, and IFN‐γ levels in co‐culture supernatants (*n* = 3). (F–J) WT or *Retreg1^−/−^* BMDCs were primed with LPS (1 µg/mL) or PBS for 24 h, followed by subsequent experiments. (F) Flow cytometry analysis of CD80, CD86, and MHC‐II expression levels (*n* = 3). (G) ELISA analysis of IL‐12 p70 levels in culture supernatants (*n* = 3). H CCK8 assay determining the proliferative activity of CD4^+^ T cells co‐cultured with BMDCs (*n* = 5). (I) Flow cytometry analysis of the proportion of divided CD4^+^ T cells stained with CFSE dye (*n* = 3). (J) ELISA analysis of IL‐2, IL‐4, and IFN‐γ levels in co‐culture supernatants (*n* = 3). For data in (A–J), each sample was assayed in technical triplicate, with mean values representing that sample. Data in (A–J) are presented as mean ± SD. Statistical analysis in panels (A–J) was performed using two‐way ANOVA with Tukey's post hoc test for multiple comparisons. ^*^ *p* < 0.05, ^**^ *p* < 0.01, ^***^ *p* < 0.001.

Surface marker analysis revealed diminished CD80, CD86, and MHC‐II expression on *Retreg1^−/−^* splenic DCs vs. WT controls following LPS exposure. (Figure 2A). Additionally, reduced production of IL‐12 was observed in *Retreg1^−/−^* mice in response to LPS stimulation (Figure 2B). CD4^+^ T cells co‐cultured with LPS‐treated *Retreg1^−/−^* DCs exhibited impaired proliferation, evidenced by decreased proportion of divided cells, indicating compromised T cell priming capacity (Figure 2C,D). IL‐2 release from T cells was decreased after co‐culturing with DCs from *Retreg1^−/−^* mice (Figure 2E). LPS‐induced IFN‐γ level in co‐culture supernatants was reduced while IL‐4 level was elevated in *Retreg1^−/−^* mice vs. controls, yielding a substantially diminished IFN‐γ/IL‐4 ratio following LPS treatment (Figure 2E).

Given TRIM13 upregulation secondary to septic insults (Figure S4A,B), paralleling RETREG1 dynamics, we explored whether LPS‐induced activation of DCs required TRIM13. *Trim13^−/−^* DCs exhibited comparable upregulation of functional markers and IL‐12 production upon LPS stimulation, with increased IL‐2 and IFN‐γ levels, and decreased IL‐4 level in co‐culture supernatants (Figure S7A–C).

To investigate whether the depletion of RETREG1 alters the maturation process of DCs, we isolated BMDCs from *Retreg1^−/−^* and WT mice and stimulated the cells with LPS for 24 h. As expected, LPS enhanced the maturation of BMDCs, as indicated by increased levels of CD80, CD86, MHC‐II, and IL‐12 (Figure 2F,G). However, *Retreg1^−/−^* BMDCs exhibited suppressed surface marker and IL‐12 expression following LPS stimulation (Figure 2F,G). CD4^+^ T cells co‐cultured with LPS‐primed WT‐BMDCs exhibited enhanced proliferation and Th1 differentiation vs. untreated controls; these responses were absent in co‐cultures with LPS‐primed *Retreg1^−/−^* BMDCs (Figure 2H–J).

These results indicate that the deficiency of *Retreg1*, but not other reticulophagy regulators (e.g., *Trim13*), specifically impairs functional activation and maturation of DCs upon LPS challenge.

### 2.3 RETREG1 Mediates Maturation and Activation of DCs in Polymicrobial Sepsis

The cecal ligation and puncture (CLP) surgery is a commonly used approach to reproduce polymicrobial sepsis (Figure S8A) [10]. We further investigated the effect of RETREG1‐mediated reticulophagy on maturation and activation of DCs in a CLP model. Splenic DCs isolated from WT mice at various post‐operative timepoints demonstrated early‐stage activation during sepsis, characterized by upregulated CD80, CD86, and MHC‐II expression with elevated IL‐12 production, consistent with ex vivo findings (Figure S8B,C). Splenic DCs isolated from CLP mice at 24 h post‐operation also promoted the immune response of T cells by inducing their proliferation as well as release of IL‐2 and IFN‐γ (Figure S8D–F). However, the function of DCs was suppressed by CLP‐induced sepsis for 72 h.

Correspondingly, RETREG1‐mediated reticulophagy increased 24 h post‐CLP, evidenced by elevated RETREG1 expression and SEC61B degradation (Figure S8G). ER‐associated autophagosomes were increased in the early stage of sepsis by confocal microscope or TEM analysis (Figure S8H,I). In contrast, RETREG1‐mediated reticulophagy in DCs was inhibited at 72 h after CLP surgery (Figure S8H,I). These findings suggest that DCs and reticulophagy exhibit similar temporal response patterns in polymicrobial sepsis.

**FIGURE 3:**
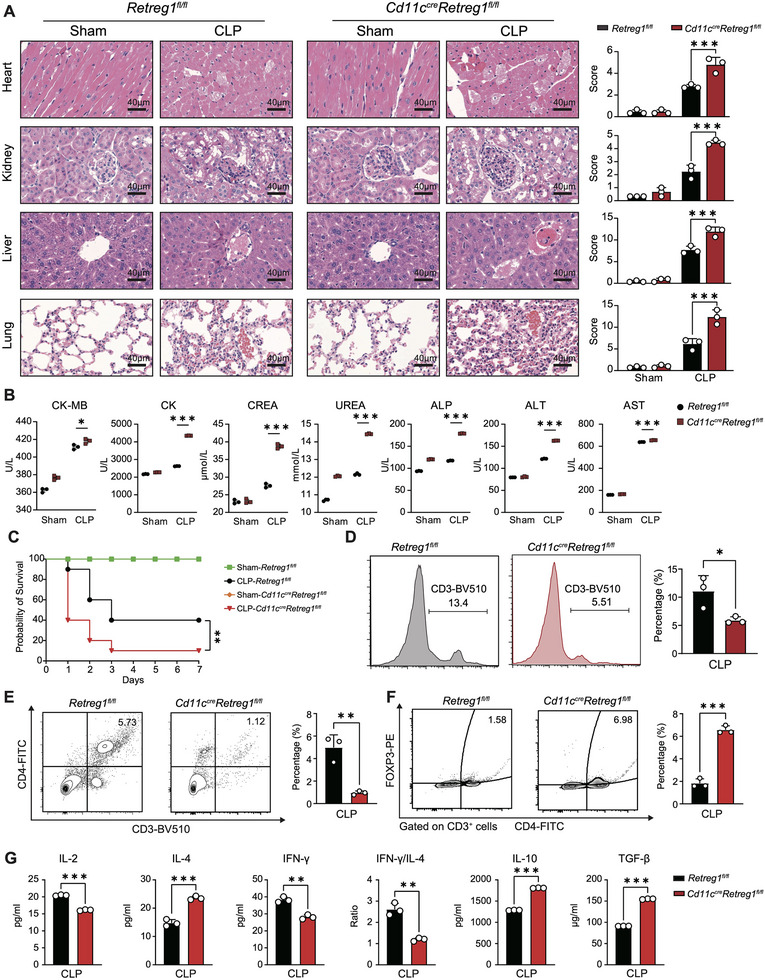
RETREG1 regulates multiple organ injuries and immune suppression in polymicrobial sepsis. PBMCs and plasma isolated from *Retreg1^fl/fl^* and *Cd11c^cre^Retreg1^fl/fl^* mice that underwent CLP or sham surgery were used for subsequent assays. (A) Representative HE images with histological scores showing pathological alterations in multiple organs, including the lung, liver, kidney, and heart (*n* = 3). (B) Biochemical analysis evaluating the functional indicators of liver (ALP, ALT, and AST), kidney (CREA and UREA), and heart (CK‐MB and CK) (*n* = 3). (C) Survival analysis of mice subjected to CLP surgery (*Retreg1^fl/fl^*‐Sham, *n* = 8; *Retreg1^fl/fl^*‐CLP, *n* = 20; *Cd11c^cre^Retreg1^fl/fl^*‐Sham, *n* = 10; *Cd11c^cre^Retreg1^fl/fl^*‐CLP, *n* = 22). (D) Flow cytometry analysis examining the percentage of CD3^+^ cells (*n* = 3). E Flow cytometry analysis of the proportion of CD3^+^CD4^+^ cells (*n* = 3). F Flow cytometry analysis assessing the proportion of CD3^+^CD4^+^Foxp3^+^ Tregs (*n* = 3). G ELISA assay of circulating levels of IL‐2, IL‐4, IL‐10, IFN‐γ, and TGF‐β (*n* = 3). Histological scoring in panel (A) five microscopic fields per specimen were evaluated and averaged. Each sample in panels (B,D–G) was measured in technical triplicate to minimize analytical bias. Data in panel (C) are representative of three independent experiments. Data in panels (A, B, D‐G) are presented as mean ± SD. Statistical analysis in panels (A,B,D–G) was performed using two‐way ANOVA with Tukey's post hoc test; survival curve comparison in panel (C) was carried out using the log‐rank test. ^*^ *p* < 0.05, ^**^ *p* < 0.01, ^***^ *p* < 0.001.

Next, we assessed the impact of RETREG1‐mediated reticulophagy on the activation of DCs in a mouse model of sepsis by using *Retreg1^fl/fl^* and *Cd11c^cre^Retreg1^fl/fl^* mice, which had conditionally depleted *Retreg1* in DCs. Splenic DCs harvested 24 h post‐CLP from *Cd11c^cre^Retreg1^fl/fl^* mice exhibited suppressed activation, evidenced by reduced maturation markers, cytokine production, and CD4^+^ T cell stimulatory capacity (Figure S9A–E). CLP induced comparable conventional dendritic cell (cDC)1 reduction and cDC2 expansion in both genotypes, with similar cDC1:cDC2 ratios, indicating *Retreg1* deficiency does not affect DC phenotypic shifts during sepsis (Figure S10A–D). Furthermore, DC‐specific *Retreg1* deletion exacerbated multi‐organ damage (heart, lung, liver, and kidney) and increased septic mortality (Figure 3A–C).

To assess the impact of *Retreg1* deficiency on sepsis‐induced immune suppression, we isolated peripheral blood mononuclear cells (PBMCs) from *Cd11c^cre^Retreg1^fl/fl^* and *Retreg1^fl/fl^* mice that underwent CLP surgery. Loss of *Retreg1* aggravated immunosuppression in CLP mice, manifested by reduced T lymphocytes and CD4^+^ T cell proportions alongside marked CD4^+^Foxp3^+^ Treg expansion (Figure 3D–F). *Cd11c^cre^Retreg1^fl/fl^* mice exhibited elevated circulating IL‐4, IL‐10, and TGF‐β levels with decreased IL‐2, IFN‐γ levels, and IFN‐γ:IL‐4 ratio following CLP (Figure 3G), supporting the role of RETREG1 as a negative regulator of sepsis‐induced immune depression.

Collectively, these studies suggest that RETREG1‐mediated reticulophagy is required for the maintenance of DC's activation and function in experimental polymicrobial sepsis.

### 2.4 Reticulophagy Inhibits Excessive ER Stress After Inflammatory Insults

Given that reticulophagy maintains cellular homeostasis by restraining ER stress overactivation [34, 35, 36], we investigated whether RETREG1‐mediated reticulophagy sustains the immune function of DCs through regulating aggravated ER stress. Western blot analysis of LPS‐stimulated splenic DCs revealed that 24‐h LPS exposure induced the phosphorylation of EIF2AK3 and eukaryotic translation initiation factor 2A (EIF2A), alongside the upregulation of heat shock protein family A member 5 (HSPA5), activating transcription factor 4 (ATF4), and DNA damage inducible transcript 3 (DDIT3), with further augmentation at 72 h (Figure S11A,B). Upregulation of ERN1 and X‐box binding protein 1 (XBP1) was observed at 24 h followed by LPS exposure, whereas no significant difference was noted at 72 h. ATF6 expression peaked at 24 h and then declined at 72 h, mirroring RETREG1 dynamics (Figure S11A,B).

**FIGURE 4:**
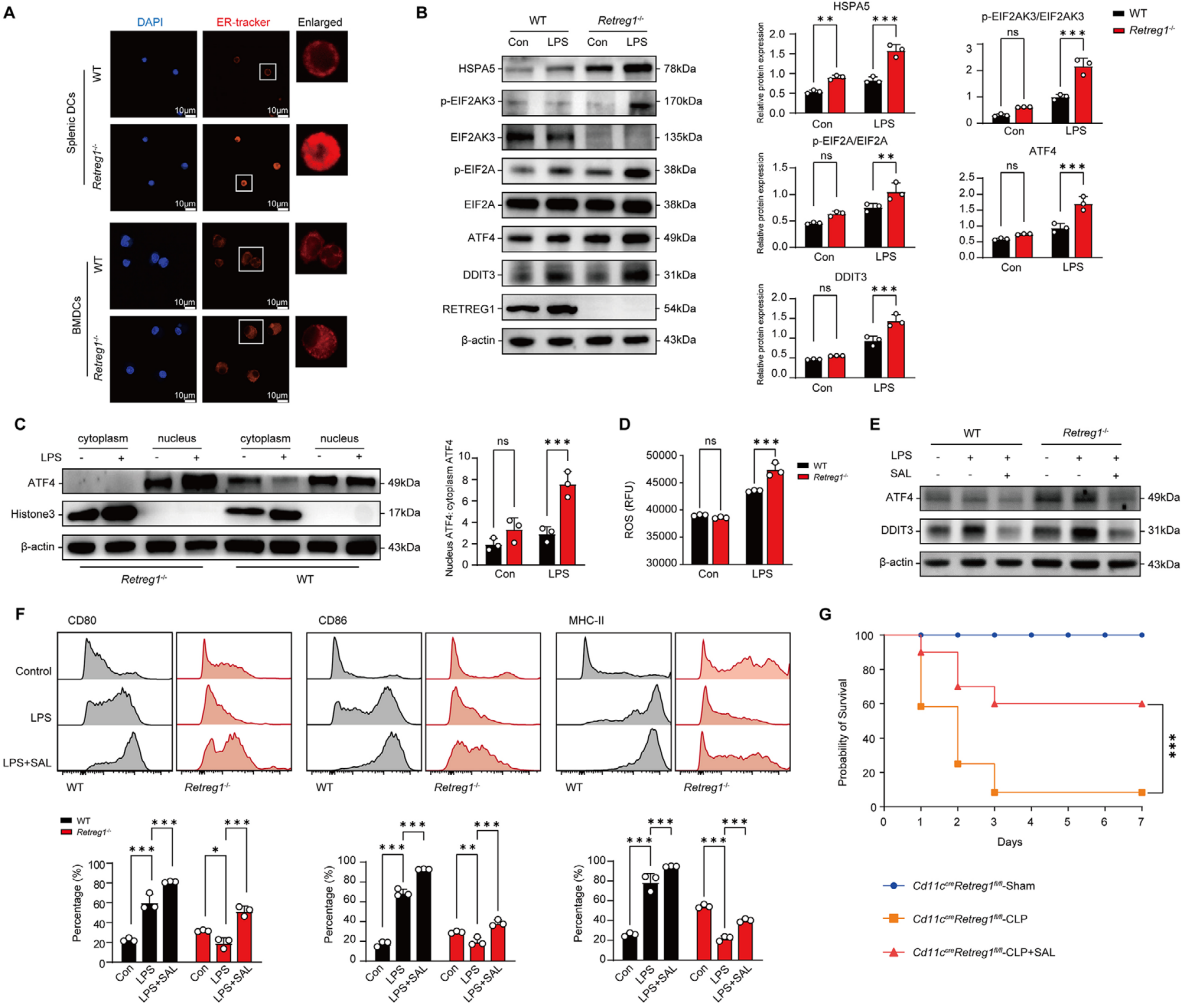
Reticulophagy inhibits excessive ER stress after inflammatory insults. (A–D) WT and *Retreg1^−/−^* DCs were treated with LPS (1 µg/mL) or PBS for 24 h. (A) Representative immunofluorescence images of ER morphology in LPS‐primed WT and *Retreg1^−/−^* splenic DCs (left panel) and BMDCs (right panel). (B) Western blot analysis indicated proteins in indicated DCs following treatment with LPS (1 µg/mL) for 24 h (*n* = 3). (C) Western blot analysis showing relative expression of ATF4 in the nucleus compared to the cytosol. β‐actin served as a control for cytosolic proteins, whereas histone 3 was used as a control for nuclear proteins (*n* = 3). (D) Intracellular ROS detection in DCs from each group (*n* = 3). E, F WT and *Retreg1^−/−^* DCs were treated with LPS (1 µg/mL) in the absence or presence of salubrinal (20 µM; SAL, a specific inhibitor of EIF2A dephosphorylation) for 24 h. (E) Western blot analysis determining expression levels of ATF4 and DDIT3. (F) Flow cytometry analysis of CD80, CD86, and MHC‐II expression levels (*n* = 3). G Survival analysis of *Cd11c^cre^Retreg1^fl/fl^* mice that underwent sham or CLP surgery, with or without intraperitoneal injection of salubrinal (20 mg/kg, 1 h post‐CLP surgery) (*Cd11c^cre^Retreg1^fl/fl^*‐Sham, *n* = 8; *Cd11c^cre^Retreg1^fl/fl^*‐CLP, *n* = 24; *Cd11c^cre^Retreg1^fl/fl^*‐CLP+SAL, *n* = 20). For data in (D,F), each sample was assayed in technical triplicate, with mean values representing that sample. Each protein sample in (B,C) was loaded onto three independent gels, and mean densitometric values were used for comparisons. Data in (A,E,G) are representative of three independent experiments. Data in panels (B–D,F) represent mean ± SD. Statistical analysis in panels (B–D,F) was performed using one‐way ANOVA and two‐way ANOVA with Tukey's post hoc test, respectively. Statistical analysis in (G) was conducted using survival curve comparison with the log‐rank test. ns, not significant; ^*^ *p* < 0.05, ^**^ *p* < 0.01, ^***^ *p* < 0.001.

Next, we examined the relationship between the EIF2AK3‐ATF4‐DDIT3 pathway and RETREG1‐mediated reticulophagy in WT and *Retreg1* ^−/−^ DCs following 24‐h LPS treatment. Immunofluorescence and TEM revealed substantial ER swelling and dilation in *Retreg1^−/−^* DCs upon LPS challenge (Figure 4A; Figure S12A,B). Consistently, LPS‐induced the upregulation of HSPA5, ATF4, DDIT3, p‐EIF2AK3, and p‐EIF2A in DCs was further exaggerated by *Retreg1* deficiency (Figure 4B), suggesting that loss of *Retreg1* in DCs augments ER stress.

ATF4 activates translational machinery genes, increasing reactive oxygen species (ROS) production and disrupting homeostasis [37]. Cellular fractionation revealed LPS‐induced nuclear ATF4 accumulation with cytoplasmic depletion, further amplified by *Retreg1* deficiency (Figure 4C). Meanwhile, intracellular ROS levels were significantly higher in *Retreg1* ^−/−^ DCs compared with those of WT‐DCs (Figure 4D). Treatment with Salubrinal, an inhibitor for EIF2A dephosphorylation [38], lowered p‐EIF2A, ATF4, and DDIT3 upregulation in LPS‐primed *Retreg1^−/−^* DCs (Figure 4E).

Salubrinal pretreatment partially rescued functional deficits in LPS‐stimulated *Retreg1* ^−/−^ splenic DCs, restoring CD80, CD86, and MHC‐II expression (Figure 4F). EIF2AK3‐ATF4‐DDIT3 pathway blockade via salubrinal attenuated multi‐organ damage and improved survival in *Cd11c^cre^Retreg1^fl/fl^* mice following CLP, confirming RETREG1‐mediated reticulophagy protects against sepsis through inhibiting ER stress (Figure 4G; Figure S13A). Intraperitoneal salubrinal administration partially reversed sepsis‐induced immunosuppression in *Cd11c^cre^Retreg1^fl/fl^* mice, evidenced by elevated T lymphocyte numbers and circulating IL‐2, IFN‐γ levels with decreased Tregs and serum IL‐10, TGF‐β levels (Figure S13B–E). Thus, RETREG1‐mediated reticulophagy alleviates ER stress in an EIF2AK3‐ATF4‐DDIT3 pathway‐dependent manner, thereby maintaining DC function in sepsis.

### 2.5 RETREG1 Regulates MHC‐II Ubiquitination in a MARCH8‐Dependent Manner

**FIGURE 5:**
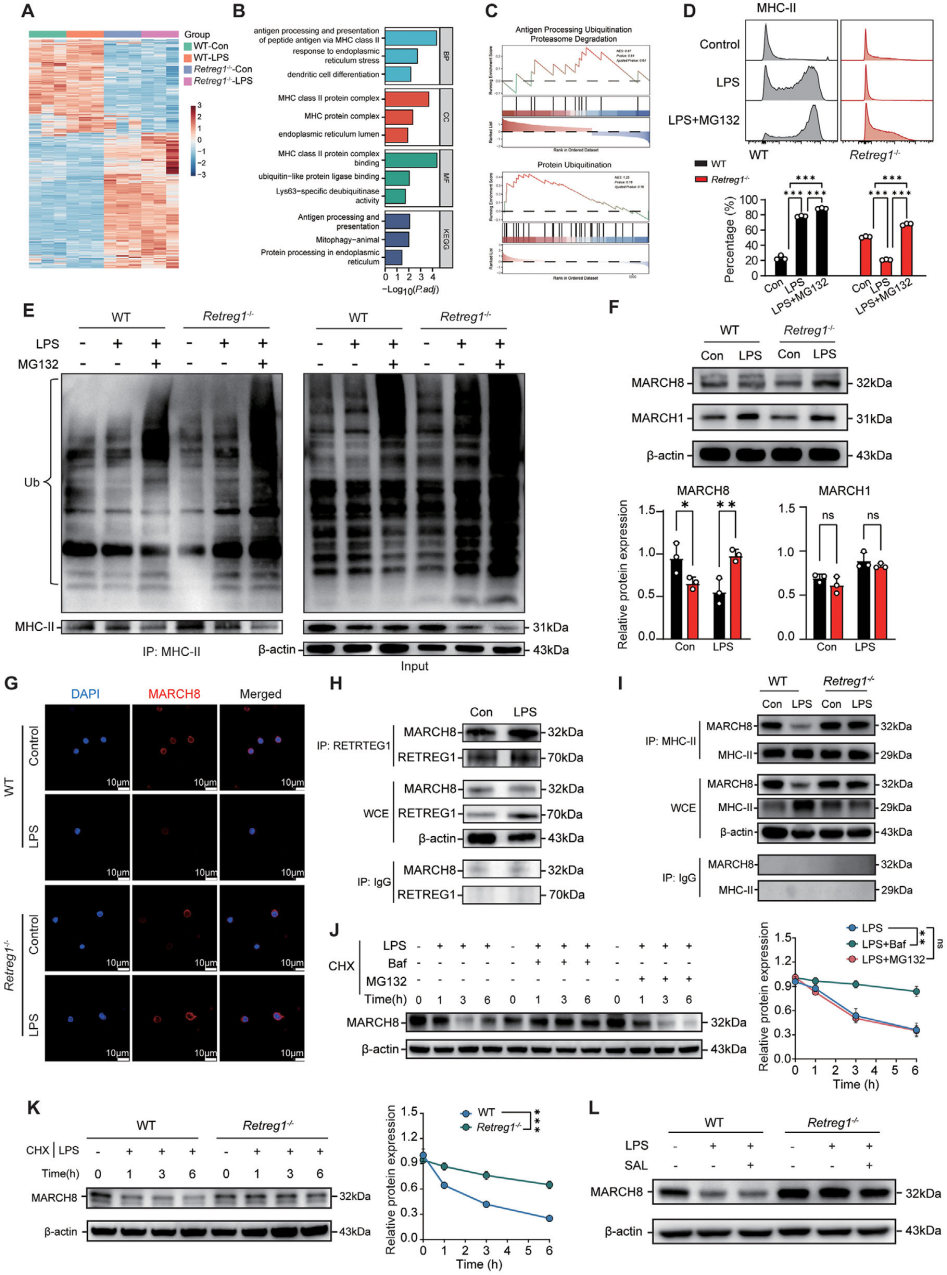
RETREG1 regulates MHC‐II ubiquitination in a MARCH8‐dependent manner. A‐C Mass spectrometry analysis was performed in WT and *Retreg1* ^−/−^ DCs treated with PBS or LPS (1 µg/mL) for 24 h. (A) Heatmap displaying the DEPs among groups. (B) Bar graph listing the enriched terms identified by GO and KEGG analyses in LPS‐treated WT and *Retreg1* ^−/−^ DCs, as shown by ‐Log_2_ (*p* value). (C) GSEA analysis of antigen processing ubiquitination proteasome degradation (upper panel) and protein ubiquitination (lower panel). (D,E) WT and *Retreg1* ^−/−^ DCs were primed with LPS (1 µg/mL for 24 h) followed by treatment of proteasome inhibitor MG132 (10 µM for 4 h prior to harvest). (D) Flow cytometry analysis assessing expression level of MHC‐II (*n* = 3). (E) Immunoprecipitation analysis detecting the ubiquitinated level of MHC‐II. (F, G) Splenic DCs collected from WT and *Retreg1* ^−/−^ mice were treated with LPS (1 µg/mL) or PBS for 24 h. (F) Western blot analysis of expression levels of MARCH1 and MARCH8 (*n* = 3). (G) Representative confocal immunofluorescence images indicating expression of MARCH8. (H) Co‐immunoprecipitation analysis of binding activity between RETREG1 and MARCH8 in LPS‐treated WT DCs. (I) Co‐immunoprecipitation analysis of binding activity between MARCH8 and MHC‐II in WT and *Retreg1*‐deficeient DCs upon LPS stimulation. (J) Immunoblotting analysis of MARCH8 expression in splenic DCs (pre‐treated with 50 µg/mL CHX to inhibit protein synthesis) stimulated with 1 µg/mL LPS for 1, 3, and 6 h, with the presence of 10 µM MG132 or autophagy inhibitor BafA1 (100 nM for 30 min prior to LPS treatment) (left panel). Time‐course analysis of MARCH8 level normalized by β‐actin (*n* = 3) (right panel). (K) Western blot (left panel) and time‐course analysis (right panel) revealing MARCH8 expression in CHX‐primed *Retreg1* ^−/−^ and WT‐DCs treated with LPS for various durations (1, 3, and 6 h) (*n* = 3). (L) Western blot analysis determining expression levels of MARCH8 in WT and *Retreg1^−/−^* DCs were treated with 1 µg/mL LPS in the absence or presence of 20 µM salubrinal for 24 h. Each sample in panel (D) was assayed in technical triplicate, with mean values representing that sample. Protein sample in (F,J,K) was loaded onto three independent gels, and mean densitometric values were used for comparisons. Data in (E,G,H,I,L) are representative of three independent experiments. Data in panels (D,F,J,K) represent mean ± SD. Two‐way ANOVA with Tukey's post hoc test (D,F) or Šidák's multiple‐comparison test (J,K) was adopted in calculating statistics. ns, not significant; ^*^ *p* < 0.05, ^**^ *p* < 0.01, and ^***^ *p* < 0.001.

Given that activated reticulophagy partially restores the downregulated expression of surface molecules on DCs, other regulatory mechanisms might be involved in maintaining DC activation in sepsis. Mass spectrometry of WT and *Retreg1^−/−^* DCs following phosphate buffer saline (PBS) or LPS treatment identified differentially expressed proteins (DEPs) visualized via heatmap (Figure 5A; Figure S14A and Table S1). The gene ontology (GO), KEGG pathway, and protein‐protein interaction (PPI) network analyses of DEPs showed that antigen processing and presentation, MHC‐II protein complex, deubiquitination activity, and ER stress were the most affected signaling cascades and components by *Retreg1* depletion following septic insults (Figure 5B; Figure S14B–G).

Gene set enrichment analysis (GSEA) analysis revealed that antigen processing, ubiquitination, proteasome degradation, and protein ubiquitination were exaggerated in LPS‐treated *Retreg1* ^−/−^ DCs compared with WT DCs (Figure 5C). Since deubiquitylation of functional molecules remains a prerequisite for functional activation and maturation of DCs, especially for MHC‐II [39, 40], we hypothesized that the suppressive expression of MHC‐II caused by *Retreg1* deletion might be due to dysregulated ubiquitination levels. MG132 treatment, a proteasome/calpain inhibitor, rescued MHC‐II expression in LPS‐treated *Retreg1^−/−^* DCs (Figure 5D).

Immunoprecipitation assays revealed decreased MHC‐II ubiquitination following LPS stimulation in WT but not *Retreg1* ^−/−^ DCs (Figure 5E). Since membrane‐associated RING‐CH‐type finger (MARCH)1/8 mediates MHC‐II ubiquitination‐dependent degradation [41, 42], we examined the regulatory role of RETREG1‐mediated reticulophagy on MARCH1/8. Western blot showed MARCH8, but not MARCH1, upregulation in LPS‐treated *Retreg1* ^−/−^ DCs (Figure 5F,G). LPS facilitated RETREG1‐MARCH8 binding and colocalization (Figure 5H). Crucially, RETREG1 regulated MARCH8‐MHC‐II interaction, with increased binding in *Retreg1*‐deficient DCs upon LPS challenge (Figure 5I). To further determine the degradation pathway of MARCH 8, we examined the expression pattern of MARCH 8 after LPS and cycloheximide (CHX) stimulation ex vivo, with the presence of inhibitors of the proteasome (MG132) or autophagy (Bafilomycin A1, BafA1). Time‐course analysis following LPS/cycloheximide treatment with MG132 or BafA1 showed rapid MARCH 8 downregulation with PBS or MG132, but more gradual decline with BafA1 (Figure 5J). Cycloheximide chase experiments demonstrated significantly prolonged MARCH 8 half‐life in *Retreg1^−/−^* DCs (Figure 5K), indicating RETREG1‐mediated autophagic MARCH8 degradation. Salubrinal treatment did not affect MARCH8 expression in either genotype, suggesting EIF2AK3‐ATF4‐DDIT3‐independent regulation (Figure 5L).

Taken together, these results indicate that RETREG1 regulates MHC‐II ubiquitination in a MARCH8‐dependent manner.

### 2.6 ATF6 Drives RETREG1 Expression and Reticulophagy Activation

**FIGURE 6:**
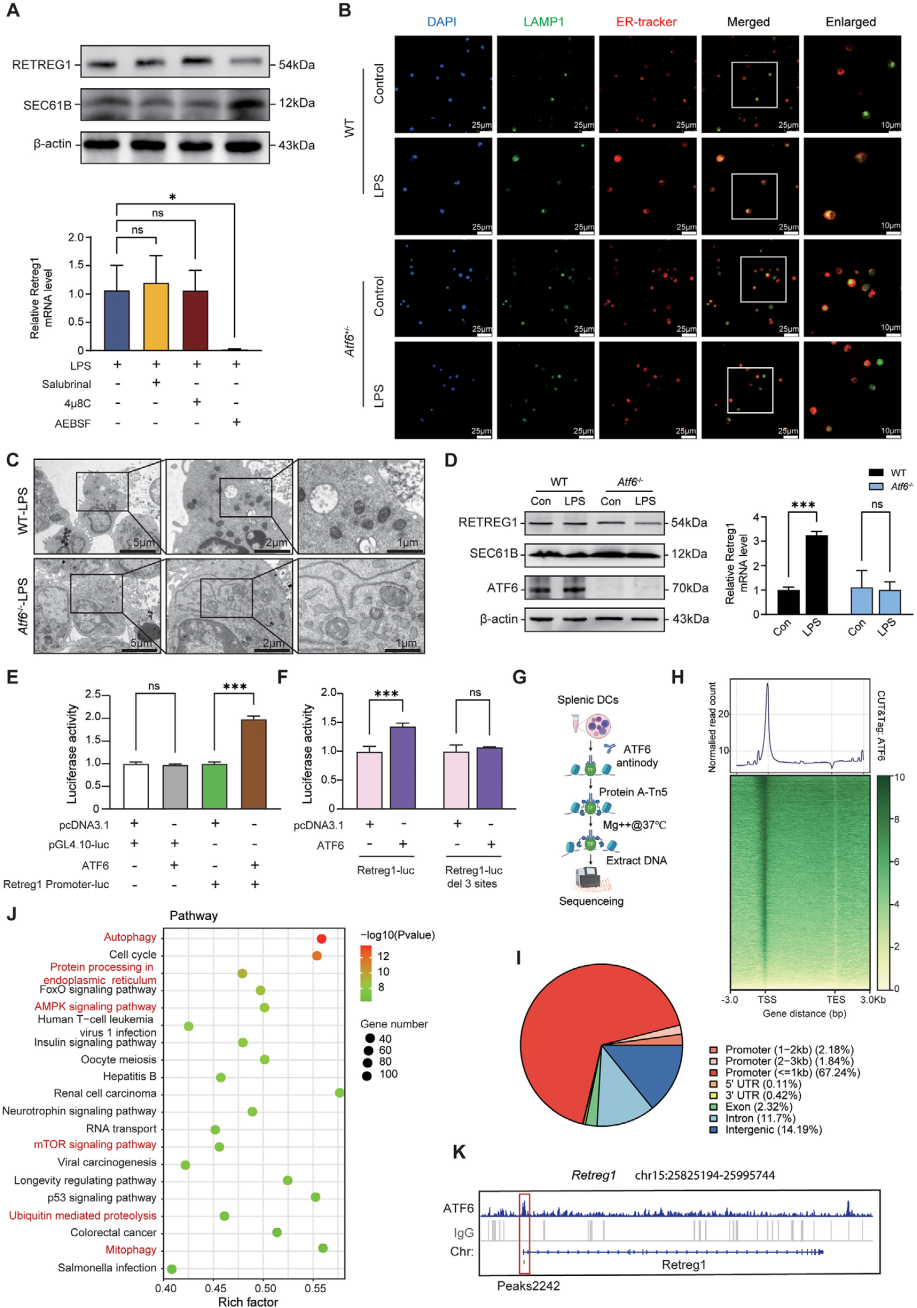
ATF6 drives RETREG1 expression and reticulophagy activation. (A) WT DCs were treated with LPS (1 µg/mL) in the absence or presence of salubrinal (20 µM; inhibitor of EIF2A dephosphorylation), 4µ8C (10 µM; an inhibitor of IRE1α) or AEBSF (100 µM; inhibitor of serine proteases) for 24 h. Western blot analysis of RETREG1 and SEC61B expressions (upper panel), and qRT‐PCR analysis quantifying relative mRNA level of *Retreg1* (lower panel) (*n* = 3). (B–D) WT and *Atf6* ^−/−^ DCs were treated with LPS (1 µg/mL) or PBS for 24 h. (B) Representative immunofluorescence images of ER colocalized with LAMP1. (C) Representative TEM images showing formation of ER‐associated autophagosomes and ER morphology. (D) Western blot analysis examining expressions of RETREG1 and SEC61B (upper panel); qRT‐PCR analysis determining mRNA level of *Retreg1* (lower panel) (*n* = 3). (E) Dual luciferase reporter gene assay indicating the binding activity of *Atf6* in the *Retreg1* promoter region (*n* = 3). (F) Luciferase activity analysis evaluating transcriptional activity of *Retreg1* promoter (Retreg1‐luc) or *Retreg1* promoter with a deletion of 3 binding sites (ZBP1‐luc del) in 293T cells overexpressing ATF6 (*n* = 3). (G–K) Sphenic DCs harvested from WT mice were subjected to CUT‐Tag experiments using anti‐ATF6 antibody. (G) Schematic diagram displaying workflow of CUT‐Tag experiment in this study. (H) Metaplot and heatmap showing distribution of ATF6 binding sites in splenic DCs. TSS, transcription start site; TES, transcription end site. (I) Pie chart exhibiting distribution of ATF6 binding regions in bound genes. (J) KEGG pathway analysis of genes predicted to be regulated by ATF6. (K) IGV analysis of genomic binding patterns of *Retreg1* locus showed the ATF6‐binding location (peaks 2242: from +267 to +724 bp, with the transcription start site as +1). For data in (A,D–F), each sample was assayed in technical triplicate, with mean values representing that sample. Data in (A,B,D) are representative of three independent experiments. Data are merged from or representative of at least two independent experiments. Data in panels (A,D–F) represent mean ± SD. Statistics were analyzed by one‐way ANOVA in (A,E) or two‐way ANOVA in (D,F) with Tukey's post hoc test. ns, not significant; ^*^ *p*< 0.05 and ^***^ *p*< 0.001.

The conventional UPR comprises three major signaling cascades: EIF2AK3‐ATF4, ERN1‐XBP1, and ATF6, each maintaining context‐dependent ER homeostasis [13, 43]. Pharmacological inhibition revealed that AEBSF‐mediated ATF6 blockade downregulated RETREG1 mRNA and protein in LPS‐stimulated DCs, whereas EIF2AK3‐ATF4 or ERN1‐XBP1 pathway inhibition had no effect (Figure 6A). Essential role of ATF6 in RETREG1 expression and reticulophagy activation was confirmed using LPS‐treated DCs from *Atf6^−/−^* mice (Figure 6B–D).

To determine whether ATF6 acts as a transcription factor for RETREG1 by directly binding to its promoter region, we performed a dual luciferase reporter gene assay in 293T cells by constructing plasmids expressing ATF6 and RETREG1 promoters (Figure S15A,B). Reporter assays confirmed direct ATF6 binding to the RETREG1 promoter (Figure 6E). In parallel, bioinformatic analysis identified three potential binding sites for ATF6 within the RETREG1 promoter region (Figure S15C) [44]. Deletion of these sites abolished ATF6‐mediated RETREG1 transcriptional activation (Figure 6F).

To further confirm the relationship between ATF6, RETREG1, and autophagy, we performed cleavage under targets & tagmentation (CUT‐Tag) experiments on activated splenic DCs (Figure 6G). Metaplot and heatmap analyses demonstrated read enrichment at transcription start sites, typical transcription factor binding regions (Figure 6H). ATF6 binding peaks predominantly localized to promoter regions (Figure 6I; Table S2). KEGG pathway analysis of ATF6 target genes revealed enrichment for autophagy, ER protein processing, and mitophagy pathways (Figure 6J). Integrative genomics viewer (IGV) analysis further identified ATF6 binding peaks in the promoter region of Retreg1 (Figure 6K; Table S2). Functional analysis showed that *Atf6^−/−^* DCs had impaired DC activation to LPS compared to WT DCs (Figure S16A–D). Correspondingly, *Atf6* deficiency led to exacerbated sepsis‐induced immune suppression, aggravated organ damage, and decreased survival in animals (Figure S17A–F).

Altogether, these results support that ATF6 is a key transcription factor for RETREG1 expression in mediating reticulophagy.

### 2.7 Deficit of Reticulophagy is Associated With the Immunosuppression of Septic Patients

**FIGURE 7:**
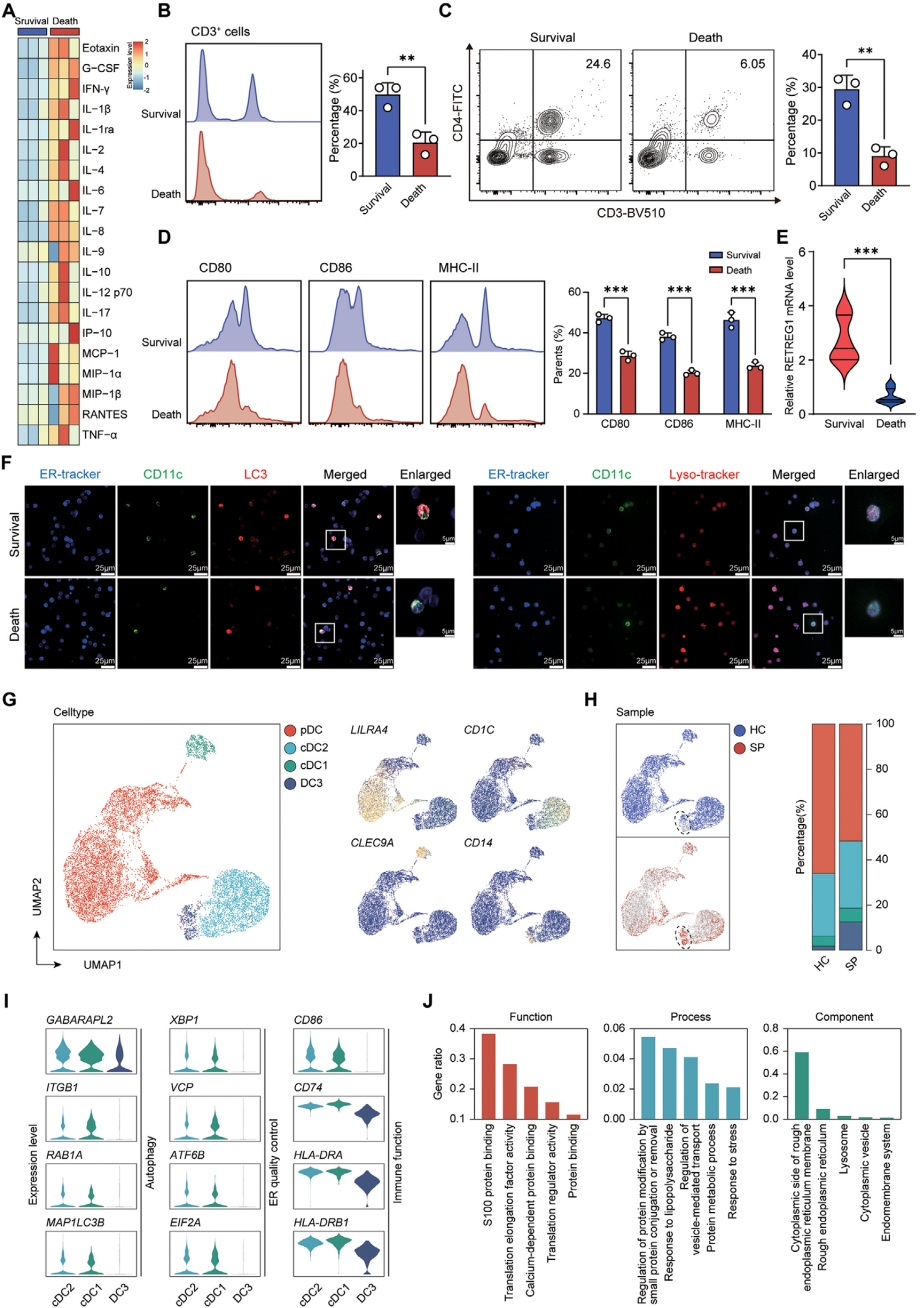
Deficit of reticulophagy is associated with the immunosuppression of septic patients. A‐F Three survivors and three non‐survivors diagnosed with sepsis were enrolled in the current study, followed by isolation of PBMCs and enrichment of circulating DCs. (A) Heatmap displaying luminex liquid suspension chip analysis of various cytokines/chemokines in plasma (*n* = 3). (B) Flow cytometry analysis examining percentage of CD3^+^ cells in PBMCs (*n* = 3). (C) Flow cytometry analysis of proportion of CD3^+^CD4^+^ cells (*n* = 3). (D) Histogram with quantitative bar charts displaying expression levels of functional markers on circulating DCs, including CD80, CD86, and MHC‐II (*n* = 3). (E) qRT‐PCR analysis of relative *RETREG1* mRNA level in circulating DCs (*n* = 3). (F) Representative confocal immunofluorescence images of CD11c and ER colocalizing with autophagosomes (left panel) as well as lysosomes (right panel). (G–J) A publicly available scRNA‐seq dataset (https://singlecell.broadinstitute. org/, SCP548) containing enriched circulating DCs was subjected to subsequent analysis. (G) Unbiased Seurat‐based clustering analysis of circulating DCs derived from healthy controls (HC: *n* = 19) and septic patients (SP: *n* = 29), generating four disparate clusters of DCs as visualized by UMAP plot (left panel); Phenotypic annotations of each DC subtype presented in independent UMAP plots based on the relative expression of cell‐type specific genes: pDC (*LILRA4*), cDC1 (*CLEC9A*), cDC2 (*CD1C*), and DC3/monocyte‐derived DC (mo‐DC) (*CD14*) (blue, low expression; yellow, high expression) (right panel). (H) UMAPs visualization of scRNA‐seq profiles according to clinical entities (left panel); Histogram showing proportion of each DC subpopulation between HC and SP (SP vs. HC: ∼7‐fold increase, 1.75% to 12.68%) (right panel). (I) Violin plots exhibiting expression levels of genes related to autophagy, ER, and DC activation. (J) Bar graph showing the enriched GO terms across DC subtypes. For data in (B–E), each sample was assayed in technical triplicate, with mean values representing that sample. Data in (F) are representative of three independent experiments. Data in panels (B–D) are presented as mean ± SD. Statistical analysis in panels (B–E) was performed using unpaired Student's t‐test. ^**^ *p* < 0.01 and ^***^ *p* < 0.001.

To validate our findings in human sepsis, we investigated whether RETREG1‐mediated reticulophagy was altered in circulating DCs by incorporating a cohort of septic patients based on Sepsis‐3 criteria, including three survivors and three non‐survivors. The demographic and clinical characteristics of the six septic patients were summarized in Table S3. The sequential organ failure assessment (SOFA) scores and serum levels of inflammatory cytokines in the non‐survivors were higher than those of the survivors (Figure 7A; Table S3). Flow cytometry revealed pronounced immunosuppression in non‐survivors vs. survivors (Figure 7B,C). Circulating DCs from non‐survivors expressed lower CD80, CD86, and MHC‐II (Figure 7D). Non‐survivors demonstrated reduced reticulophagic activity in peripheral blood DCs, evidenced by decreased *RETREG1* mRNA and diminished CD11c/ER colocalization with autophagosomes and lysosomes (Figure 7E,F).

We further analyzed a publicly available single‐cell RNA‐seq dataset containing CD45^+^ cells and enriched circulating DCs from three clinical entities: urinary tract infection (UTI) with or without organ dysfunction, bacterial sepsis in hospital wards, and ICU admission with or without sepsis [45]. Integration of circulating DCs from healthy controls and septic patients revealed 7 UMAP‐defined clusters annotated by canonical markers (Figure 7G; Figure S18A, Tables S4 and S5). Septic patients exhibited plasmacytoid dendritic cell (pDC) and cDC depletion with substantial DC3 enrichment (Figure 7H; Table S6). Pseudotime trajectory analysis positioned DC3 at late developmental stages, suggesting cDC1/cDC2 origin during sepsis (Figure S18B). Gene expression profiling revealed concurrent downregulation of reticulophagy and activation genes specifically in DC3, confirmed by pseudotime heatmap analysis (Figure 7I,J; Figure S18B).

These results suggest that sepsis‐induced DC dysfunction can be attributed to a deficit in reticulophagy, thereby leading to immune suppression and worsening clinical outcomes in septic patients.

## 3 Discussion

Sepsis occurs when an infection exceeds local tissue containment, inducing a cascade of dysregulated immune response and subsequent multiple organ failure. In this study, we elucidated the critical role of RETREG1 in sustaining the maturation and function of DCs during sepsis through the mediation of reticulophagy. Specifically, RETREG1‐mediated reticulophagy mitigated excessive ER stress via the EIF2AK3‐ATF4‐DDIT3 pathway. It regulated MHC‐II expression in DCs by degrading MARCH 8, which governs MHC‐II ubiquitination and trafficking. We also demonstrated that ATF6 transcriptionally regulated RETREG1 expression in activated DCs, representing an upstream signaling pathway for reticulophagy activation in DCs (Figure S19).

Functional collapse of DCs is a hallmark of sepsis‐induced immunosuppression, contributing to poor clinical outcomes in sepsis patients [46, 47]. Therefore, elucidating the precise mechanisms underlying DC dysfunction may help reverse sepsis‐related immune paralysis [48, 49]. ER homeostasis is critical for immune cell survival and function in sepsis, making targeting excessive ER stress a novel therapeutic avenue for infectious diseases [16, 17, 18, 50, 51, 52]. For instance, ER stress is critically involved in the maturation and activation of DCs in response to damage‐associated molecular patterns, such as high mobility group box‐1 protein [53]. However, persistent activation of ER stress can cause apoptosis of immune cells, including DCs [18, 43, 54]. In contrast, the activation of the UPR or autophagy, especially reticulophagy, can mitigate ER stress overactivation by promoting the degradation of unfolded proteins and reducing damaged ER, thereby maintaining immune cell function [29, 55, 56, 57].

Our findings suggest that RETREG1‐mediated reticulophagy is crucial for the maturation and functional activation of DCs both in vitro and in vivo, although other autophagy receptors or selective autophagy processes may exert synergistic actions. For example, activation of PTEN‐induced putative kinase 1‐dependent mitophagy reverses LPS‐ and IFN‐γ‐induced macrophage activation, leading to immunosuppression, impaired bacterial clearance, and reduced survival in murine sepsis models [58]. Conversely, other studies have shown that impaired mitophagy accelerates death from septic shock [59, 60], suggesting that the impact of selective autophagy on host immune responses may be context‐dependent.

Our results showed augmented ER stress upon prolonged septic exposure, associated with reduced reticulophagic activity and impaired DC function. Sestrin2 deficiency resulted in overactivation of ER stress and immune suppression in DCs following septic challenge, which could be partially rescued by inhibiting the EIF2AK3‐ATF4‐DDIT3 pathway [11]. Similarly, other studies have reported that blocking the EIF2AK3 pathway in DCs restores effector CD4^+^ T cell responses and reduces airway inflammation caused by respiratory syncytial virus infection [61]. Our data suggests that *Retreg1* deficiency leads to hyperactivation of ER stress, as evidenced by overactivation of the EIF2AK3‐ATF4‐DDIT3 pathway, ER fragmentation, and ROS overproduction. Consistent with our previous findings [11], salubrinal administration, which dephosphorylates EIF2A, attenuated DC functional deficits in sepsis. These results indicate that RETREG1‐mediated reticulophagy inhibits ROS overproduction associated with upregulated EIF2AK3 signaling [62].

The expression of co‐stimulatory markers on antigen‐presenting cells, including MHC‐II, CD80, and CD86, is critical for priming immune response against pathogen invasion. This process can be compromised due to ER dysfunction, as the ER is the primary site for processing and transporting surface‐resident molecules [52]. The autophagy machinery and the ubiquitin‐proteasome system regulate MHC‐I/II antigen presentation by modulating their surface expression [63, 64]. We found that the repressive expression of MHC‐II induced by *Retreg1* deficiency could be reversed by inhibiting the proteasome, indicating dysregulated ubiquitination. Furthermore, impaired MARCH8 degradation due to RETREG1‐mediated reticulophagy defects resulted in the downregulation of MHC‐II. These findings implicate an important interaction between selective autophagy and the UPR in regulating DC function, while the exact relationship between MARCH 8 expression and selective autophagy requires further investigation.

Many transcription factors are activated during the ER stress response. Among them, the ATF/cyclic AMP response element‐binding family plays a cell type‐dependent role in shaping ER stress [65, 66]. ATF4 links ER stress to RETREG1‐ and TEX264‐mediated reticulophagy in glioblastoma cells [28]. However, treatment with inhibitors and knockout animal experiments showed that ATF6, but not ATF4, directly regulated RETREG1 expression and reticulophagy activation in DCs. The CUT&Tag experiments further highlighted the role of ATF6 in regulating genes associated with autophagy and selective autophagy pathways, including mitophagy, protein kinase AMP‐activated catalytic subunit alpha 2, and mechanistic target of rapamycin signaling pathways. It has been indicated that ATF6 broadly coordinates ER stress and autophagy‐related pathways by activating multiple autophagy‐associated genes, such as *LC3*, *ATG3*, *ATG9*, and *ATG12* [67, 68]. Further understanding of the transcriptional cofactors or repressors of ATF6 may provide additional strategies for targeting ER stress‐related autophagic responses.

Several limitations should be noted when interpreting our findings. First, RETREG1 has been demonstrated to maintain ER homeostasis under both basal and stress conditions, the absence of which results in extensive ER stress and subsequent cell death, involved in various pathophysiological processes [69]. Nevertheless, it is unclear whether other RETREG paralogues (e.g., RETREG2 and RETREG3) that exhibit attenuated capacity in remodeling the ER network under basal conditions and act in concert with RETREG1 activity as facilitators, play similar roles in regulating DC‐mediated immunity [70]. Second, although the current study suggests that RETREG1 is critically involved in reticulophagy, a reticulophagy‐independent function of RETREG1 in modulating DC maturation and function cannot be ruled out. Moreover, we demonstrated the role of RETREG1 in regulating DC function via MARCH8‐dependent MHC‐II ubiquitination, while co‐stimulatory molecules, including CD80 and CD86, were not interrogated in this study, both of which were also reportedly represent the substrates of MARCH8. Third, *Atf6* conditional knockout mice are needed to determine the distinct cellular or tissue‐derived impacts on regulating immune responses in sepsis. Finally, the relatively small sample size of the clinical cohort impairs the generalizability of our findings in human sepsis, which is insufficient to establish a causal relationship between reticulophagic activity in DCs and clinical prognosis among septic patients. In addition, distinct pathophysiological features between LPS/CLP treatment in rodents and polymicrobial sepsis in patients are non‐neglectable, since laboratory settings merely mimic the acute phase of septic challenge, but are incapable of simulating the delayed immune response elicited in septic patients who receive sustained intensive care during hospitalization. Therefore, further studies are needed to investigate whether targeting RETREG1 can protect patients from sepsis‐induced immune paralysis.

## 4 Conclusion

In summary, we demonstrate that ATF6‐dependent RETREG1 expression controls DC maturation and function through reticulophagy during sepsis. These findings provide valuable insights into the cellular and molecular mechanisms underlying sepsis‐induced immunosuppression, potentially guiding the development of new therapeutic strategies for septic complications.

## 5 Experimental Section

### 5.1 Mice

WT C57BL/6J mice aged 6–8 weeks (20–25 g) were purchased from the Laboratory Animal Science of Chinese Academy of Medical Sciences, Beijing, China. *Retreg1* ^−/−^ mice were generated and supplied by Shanghai Model Organisms Center, Shanghai, China. Conditional knockout mice for *Retreg1* in DCs (*Cd11c^cre^Retreg1^fl/fl^*) and their littermate controls (*Retreg1^fl/fl^*), as well as conditional knockout mice for *Trim13* in DCs (*Cd11c^cre^Trim13^fl/fl^*) and their littermate controls (*Trim13^fl/fl^*), and *Atf6* ^−/−^ mice were purchased from Cyagen Transgenic Animal Center, Guangzhou, China. All mice were maintained under specific pathogen‐free conditions with a 12‐h light/dark cycle. Only male mice were used for the experiments. All experimental procedures were conducted in accordance with the National Institutes of Health Guide for the Care and Use of Laboratory Animals and were approved by the Scientific Investigation Board of Chinese PLA General Hospital (Approval No. 2022‐X18‐84).

### 5.2 Polymicrobial Sepsis Model

The murine model of polymicrobial sepsis was established using CLP surgery [71, 72]. Mice were anesthetized with an intraperitoneal injection of pentobarbital sodium (80 mg/kg body weight) and placed supine. The abdominal skin was disinfected, and a 1 cm midline incision was made to expose the cecum. The cecum was ligated below the ileocecal valve and punctured once with a 21‐gauge needle. A small amount of feces was extruded by gentle compression of the ligated cecum before returning it to the abdominal cavity. The incision was then closed. All mice received fluid resuscitation via subcutaneous injection of 1 mL of 0.9% normal saline. In the sham group, mice underwent identical procedures except for the ligation and puncture of the cecum. Survival rates were monitored for 7 days post‐CLP surgery.

### 5.3 Isolation of Splenic DCs and Preparation of BMDCs

Murine spleens were harvested and dispersed in precooled PBS. Splenocytes were collected using a 70 µm cell strainer. Splenic mononuclear cells were isolated by Ficoll‐Paque density gradient centrifugation (3000 rpm, 15 min). Following the manufacturer's instructions, splenic DCs were separated from the mononuclear cells using a magnetic cell sorting system (Miltenyi Biotech, Bergisch Gladbach, Germany, #130‐125‐835). Mononuclear cells were incubated with CD11c^+^ microbeads (10 µL per 10^7^ cells) at 4°C for 15 min, and splenic DCs were then collected via magnetic separation with MS columns.

To obtain BMDCs, bone marrow cells were flushed from the femur and tibia of C57BL/6 mice and passed through a 70 µm cell strainer for disaggregation. The cells were washed and resuspended at 1 × 10^6^ cells/mL in Roswell Park Memorial Institute (RPMI) 1640 medium (Solarbio, Beijing, China, #31800) supplemented with 10% heat‐inactivated fetal bovine serum (FBS; Gibco/Life Technologies, Grand Island, NY, # A5670801), 100 U/mL penicillin, and 100 mg/mL streptomycin (Gibco/Life Technologies, Grand Island, NY, #15070063). The cell suspension was added to Ultra‐Low Attachment Culture Dishes (Corning Inc., Corning, NY, #4615) at 20 mL per dish, and 20 ng/mL granulocyte‐macrophage colony‐stimulating factor (GM‐CSF) (PeproTech, Rocky Hill, NJ, #250‐05) was added. The culture medium was refreshed on days 3, 6, and 8. After 8 days of differentiation, BMDCs were collected for subsequent experiments.

### 5.4 Cell Culture and Stimulation

Splenic DCs and BMDCs were cultured in complete RPMI 1640 medium in a 5% CO_2_, 37°C humidified incubator. Cells were incubated for 24 h before all experiments. HEK293T cells were cultured in Dulbecco's Modified Eagle's Medium (DMEM) (Solarbio, Beijing, China, #11995) supplemented with 10% FBS, 100 U/mL penicillin, and 100 mg/mL streptomycin in a 5% CO_2_ humidified incubator.

For in vitro experiments, splenic DCs and BMDCs were stimulated with LPS (1 µg/mL; Sigma–Aldrich, St. Louis, MO, #L4516) and collected at various time points. To inhibit the three pathways of the UPR, salubrinal, 4µ8C, and AEBSF (Selleck Chemicals, Shanghai, China, #S2923, #S7272, and #S7378) were administered before LPS stimulation. Z‐VAD‐FMK (zVAD, Selleck Chemicals, Shanghai, China, #S7023) was employed to inhibit apoptotic activity, supplemented to the culture medium 30 min before LPS administration. MG132 (Selleck Chemicals, Shanghai, China, #S2619) was used as a proteasomal inhibitor, added to the culture medium 4 h before cell collection. Bafilomycin A1 (BafA1, Santa Cruz Biotechnology, sc‐201550A, Dallas, TX) was adopted to block autophagic degradation, which was treated 30 min before LPS stimulation. To inhibit protein synthesis, cells were primed with cycloheximide (CHX, Sigma‐Aldrich, St. Louis, MO, #239764) 30 min before LPS treatment.

### 5.5 CUT‐Tag

The cell suspension was washed twice with wash buffer, then coated with concanavalin A magnetic beads (Bangs Laboratories, Shanghai, China) at room temperature for 10 min. The bead‐bound cells were washed and incubated overnight at 4°C with either the primary antibody (anti‐ATF6 antibody: Abcam, ab22783, Cambridge, MA) or an IgG control antibody (normal rabbit IgG: Millipore, 12–370, Darmstadt, Germany) at a 1:50 dilution. Following this, cells were incubated with a secondary antibody (anti‐Rabbit IgG antibody, Goat monoclonal: Millipore, AP132, Darmstadt, Germany). A pA‐Tn5 adapter complex at a 1:100 dilution was then added to the washed cells and incubated at room temperature for 1 h. The cells were resuspended in tagmentation buffer and incubated at 37°C for 1 h. DNA was purified using phenol‐chloroform‐isoamyl alcohol extraction and ethanol precipitation. To amplify the library, DNA was mixed with a universal i5 primer and a uniquely barcoded i7 primer, followed by the addition of NEBNext HiFi 2 × PCR Master Mix. Samples were then placed in a thermocycler under recommended cycling conditions. The size distribution of the library was determined using Agilent 4200 TapeStation analysis. DNA sequencing was performed on the Illumina NovaSeq 6000 platform (Illumina, San Diego, CA).

### 5.6 Preparation of Human Samples

Blood samples from septic patients were collected at ICU admission from the First Medical Center of Chinese PLA General Hospital, before any treatments. This study was approved by the Institutional Review Board of the First Medical Center of Chinese PLA General Hospital (Approval No. S2022‐735‐01), and written informed consent was obtained from each patient. The cohort was part of an ongoing clinical trial registered in the Chinese Clinical Trial Registry (ChiCTR, https://www.chictr.org.cn/, registration number: ChiCTR2300071841).

Sepsis was diagnosed according to the Sepsis 3.0 criteria (Sequential Organ Failure Assessment [SOFA] score ≥ 2 along with confirmed or suspected infection) [1]. Clinical and demographic data at ICU admission were collected using a predesigned data collection form, which included age, gender, comorbidities, source of infection, laboratory findings, and in‐hospital mortality. The SOFA score was calculated within the first 24 h after ICU admission, using the value associated with the greatest severity of illness.

### 5.7 Statistical Analysis

All data were presented as mean ± standard deviation (SD). *p*‐values were determined using the unpaired Student's *t*‐test, one‐way ANOVA, or two‐way ANOVA, as appropriate. Tukey's and Šidák's multiple‐comparisons tests and the LSD‐*t* test were employed for multiple comparisons. Kaplan‐Meier analysis was conducted for survival rates, and the log‐rank test was used to assess differences between survival curves. Two‐tailed *p*‐values less than 0.05 were considered statistically significant. The aforementioned analyses were performed using SPSS software version 23.0 (SPSS Inc., Chicago, IL) and GraphPad Prism 8. No statistical methods were applied to pre‐determine sample sizes, which were in line with historical data or previous experience in similar experimental settings.

## Author Contributions

Y.M.Y., D.L.T., Z.Z., H.J.K., C.R., and R.Q.Y. conceived the study. R.Q.Y., C.R., L.Y.Z., J.Y.L., W.F.W., Y.X.L., L.X.W., and Y.D. performed the experiments. P.Y.H., P.Y.Z., S.T., T.Z., M.Y.W., S.T.W., N.D., Y.W., H.Z., X.M.Z., Z.C.Z., and G.S.W. developed the reagents and analytical tools necessary for the study. L.W. and S.Q.L. provided clinical samples. Z.X.L. and T.Z. conducted the bioinformatic analyses. R.Q.Y. and C.R. drafted the initial version of the manuscript. R.Q.Y., L.Y.Z., and C.R. analyzed the data. Y.M.Y., D.L.T., Z.Z., H.J.K., X.H.D., and Z.F.X. supervised the study and provided critical revisions of the manuscript. All authors read and approved the final draft for publication.

## Funding

This work was supported by grants from the National Natural Science Foundation of China (82241062, 82130062, 82272187, 82402536), the National Key Research and Development Program of China (2022YFA1104600), the Beijing Natural Science Foundation (7244296), the Beijing Hospitals Authority Youth Programme (QML20230309), the Beijing Physician Scientist Training Project (BJPSTP‐2024‐24), and the Chinese Postdoctoral Science Foundation Grant (2019M664003).

## Ethics Statement

This Study Involving Human Participants Was Reviewed and Approved By the Institutional Review Board of the First Medical Center of Chinese PLA General Hospital (Approval No. S2022‐735‐01), and Written Informed Consent Was Obtained from each patient. All animal Experimental Procedures Were Conducted in Accordance With the National Institutes of Health Guide for the Care and Use of Laboratory Animals and Were Approved By the Scientific Investigation Board of Chinese PLA General Hospital (Approval No. 2022‐X18‐84).

## Clinical Trial Registration

The cohort was part of an ongoing clinical trial registered in the Chinese Clinical Trial Registry (ChiCTR, https://www.chictr.org.cn/, registration number: ChiCTR2300071841).

## Conflicts of Interest

The authors declare no conflicts of interest.

## Supporting information




**Supporting File**: advs74811‐sup‐0001‐SuppMat.docx.

## Data Availability

The data that support the findings of this study are available in the supplementary material of this article.
