# Supplementary material for: RETREG1‐Mediated Reticulophagy is Essential for Dendritic Cell Maturation and Function in Sepsis
Source: Adv Sci (Weinh). 2026 Mar 24;13(29):e11021. doi: 10.1002/advs.202511021 (PMC13205583; doi:10.1002/advs.202511021)
Supplement: Supplementary file 1 — Supporting File: advs74811‐sup‐0001‐SuppMat.docx. [file ADVS-13-e11021-s001.docx]

**Supporting Information**

**RETREG1-mediated Reticulophagy is Essential for Dendritic Cell Maturation and Function in Sepsis**

*Ren-qi Yao, Chao Ren, Li-yu Zheng, Jing-yan Li, Wen-feng Wu, Yu-xuan Li, Li-xue Wang , Yu Duan, Lu Wang, Shuang-qing Liu, Peng-yi He, Peng-yue Zhao, Sen Tong, Zhi-xuan Li, Tuo Zhang, Meng-yao Wu, Shu-ting Wei, Ning Dong, Yao Wu, Hui Zhang, Xiao-mei Zhu, Zi-cheng Zhang, Guo-sheng Wu, Zhao-fan Xia,* *Xiao-hui Du, Hong-jun Kang**,* *Zui Zou, Dao-lin Tang, Yong-ming Yao*

Correspondence to: [c_ff@sina.com](mailto:c_ff@sina.com) (Y.Y.), daolin.tang@utsouthwestern.edu (D.T.), zouzui@smmu.edu.cn (Z. Z.), and [doctorkang301@163.com](mailto:doctorkang301@163.com) (H.K.).

**Experimental Section**

***Isolation of splenic T lymphocytes and proliferative assay***

Splenic CD4^+^ T lymphocytes were isolated from mononuclear cells using a magnetic cell sorting system (Miltenyi Biotech, Bergisch Gladbach, Germany, #130-104-454) following the manufacturer’s instructions. Briefly, mononuclear cells were incubated with CD4^+^ microbeads (20 μL per 10^7^ cells) for 15 min at 4°C, and CD4^+^ T lymphocytes were collected via magnetic separation. The isolated CD4^+^ T lymphocytes were resuspended in complete RPMI 1640 medium (10% FBS, 100 U/mL penicillin, and 100 μg/mL streptomycin) and seeded into 96-well plates at a density of 4×10^5^ cells per well. They were cultured in the presence of soluble CD3 (1 μg/mL) and CD28 (1 μg/mL) antibodies (BioLegend, San Diego, CA, #155702, #117003) for 24 h in a 5% CO_2_, 37°C incubator.

Subsequently, splenic DCs were added to the wells at a DC cell ratio of 1:20, followed by a 72-hour co-culture. The proliferative activity of CD4^+^ T lymphocytes was assessed using the CCK8 kit (Dojindo Laboratories, Kumamoto, Japan, #CK04) and the CFSE Cell Division Tracker Kit (BioLegend, San Diego, CA, #423801) according to the manufacturer’s protocols. For the CCK8 assay, 10 μL of CCK8 solution was added to the co-culture system and incubated for 2 h. The optical density was measured at 450 nm using an ELISA plate reader (Spectra MR, Dynex, Richfield, MN). For CFSE staining, CD4^+^ T cells were labeled with CFSE dye as per the manufacturer’s instructions, followed by activation and co-culture with splenic DCs or BMDCs. CD4^+^ T cells were harvested on day 4 and analyzed by flow cytometry.

***Isolation of murine PBMCs***

Peripheral blood mononuclear cells (PBMCs) were isolated using a density gradient centrifugation-based PBMC isolation kit (TBDsciences, LTS1077, Tianjin, China) according to the manufacturer's instructions. Briefly, whole blood (700-800 μL per mouse) was collected from murine retro-orbital bleeding using sodium heparin blood collection tubes and diluted with precooled PBS containing EDTA. The diluted blood was layered over Lymphoprep and centrifuged for 30 min at 450 g, after which the PBMC-containing interphase was collected. Red blood cells were then lysed with a lysis buffer for 5 min, and the reaction was terminated by adding RPMI supplemented with 2% FBS. The cells were washed and resuspended in PBS containing bovine serum albumin (BSA) for further assays.

***Measurement of cytokine levels***

Cell culture supernatants were assayed using ELISA kits (Excell Inc., Shanghai, China) to quantify the concentrations of IL-2, IL-4, IL-10, IL-12, TGF-β, and IFN-γ, following the manufacturer's protocols. The results were assessed and analyzed with an ELISA plate reader (Spectra MR, Dynex, Richfield, MN). To quantify the levels of various cytokines and chemokines in plasma derived from septic patients, Luminex liquid suspension chip detection was employed. The Bio-Plex Pro™ Human Cytokine Screening Panel 23-plex (Bio-Rad, Austin, TX) was used according to the manufacturer's instructions, and the results were analyzed using the Bio-Plex MAGPIX System (Bio-Rad, Austin, TX).

***Biochemical analysis***

Mice in each group were anesthetized *via* intraperitoneal injection of 4% pentobarbital, followed by orbital blood collection. Samples were then left at room temperature for 2 h, and centrifuged at 3000 rpm for 15 min at 4°C. The collected supernatants represented serum, which were stored at -20°C for subsequent measurement. The levels of blood alkaline phosphatase (ALP), alanine aminotransferase (ALT), aspartate aminotransferase (AST), creatine kinase (CK), creatine kinase-myocardial band (CK-MB), creatinine (CREA), and urea (UREA) were assessed using a fully automatic biochemical analyzer (Chemray 800, Leidu, Shenzhen, China).

***Histological examination***

The dissected lung, heart, liver, and kidney tissues were fixed in 4% paraformaldehyde (PFA) overnight at 4 °C and subsequently embedded in paraffin blocks. Tissue sections (4-5 μm thick) were deparaffinized and stained with hematoxylin-eosin (HE). Histological features were observed and analyzed using a microscope (Nikon Instruments Co., Japan). Two experienced histologists, blinded to the experimental groups, independently evaluated the sections. The histological score of each organ was measured on the basis of a four-point scale (0 [absent] to 3 [severe]) assigned to each criterion as previously described. Also, a minimum of three microscopic areas were examined to score each specimen.

***RNA extraction and qRT-PCR analysis***

Splenic DCs were harvested for total RNA extraction using Trizol kits (ThermoFisher Scientific, 5596026, Waltham, MA) according to the manufacturer’s instructions. The purity and concentration of RNA were measured using an ultraviolet spectrophotometer. Subsequently, 2 μg of RNA from each sample was used for reverse transcription with the Reverse Transcription System (Promega, A3500, Madison, WI), following the manufacturer’s protocol. Quantitative real-time PCR (qRT-PCR) was performed using a PCR apparatus. The qRT-PCR program was initiated at 95°C for 10 min, followed by 35 cycles of 95°C for 40 sec, 60°C for 30 sec, and 72°C for 25 sec. The Ct values of the target genes were normalized to the housekeeping gene GAPDH, and the relative mRNA expression of RETREG1 was calculated using the 2^−ΔΔCt^ method compared to the control group. The primers used were as follows: *Retreg1* (5’-TGAAGGTGAAGACTT TGGACC-3’ and 5’-AAACAATGGACACAAAAATG CAC-3’), *Gapdh* (5’-AGAAGGCTGGGGCTCATTTG-3’ and 5’-AGGGGCCA TCCACAGTCTTC-3’), *RETREG1* (5’- ACTTGGGCGTGTTATTATGC -3’ and 5’- GCCTGGGTCTTTCATCTGG -3’) and *GAPDH* (5’- CACCCACTCCTCCACCTT TGA -3’ and 5’- TCTCTCTTCCTCTTGTGCT CTTGC -3’).

***Dual luciferase reporter gene assay***

The binding interaction between ATF6 and the *Retreg1* promoter was verified using a dual luciferase reporter gene assay. The procedure involved the following steps: (1) Construction of vector plasmids: pGL4.10-MCS-luc2, pHS-AVC-LW1854 (pGL4.10-*Retreg1* Promoter, mouse, wild-type-luc2), and pHS-AVC-0820 (pGL4.10-*Retreg1* Promoter, mouse, mutant-luc2); (2) Transfection of HEK293T cells with the constructed vector plasmids; (3) Measurement of luciferase activity using the dual-luciferase reporter assay system (Promega, Madison, WI) according to the manufacturer's protocol 48 h post-transfection. Firefly luciferase activity was normalized to Renilla luciferase activity.

***Flow cytometry analysis***

The functional status and phenotypes of splenic DCs and BMDCs were determined using flow cytometry analysis. This included assessing the proportion of CD3^+^ cells, CD3^+^CD4^+^ T cells, CD4^+^Foxp3^+^ Tregs, cDC1s, and cDC2s. The antibodies used were as follows: APC-conjugated anti-mouse CD80 antibody (1:100; BioLegend, 104714, San Diego, CA), PE-conjugated anti-mouse CD86 antibody (1:100; BioLegend, 105008, San Diego, CA), FITC-conjugated anti-mouse MHC-II antibody (1:100; BioLegend, 107606, San Diego, CA), BV510-conjugated anti-mouse CD3 antibody (1:100; BioLegend, 100234, San Diego, CA), FITC-conjugated anti-mouse CD4 antibody (1:100; BioLegend, 116004, San Diego, CA), BV510-conjugated anti-mouse CD11c antibody (1:100; BioLegend, 117353, San Diego, CA), PE-conjugated anti-mouse CD8α antibody (1:100; BioLegend, 100708, San Diego, CA), FITC-conjugated anti-mouse CD172α antibody (1:100; BioLegend, 144006, San Diego, CA) and PE-conjugated anti-mouse FOXP3 antibody (1:50; Invitrogen, 12-4771-82, Carlsbad, CA).

To measure the expression levels of surface molecules, cells were stained in FACS buffer (PBS containing 2% FBS and 5 mM EDTA) with fluorochrome-conjugated antibodies for 30 minutes at 4°C. For intracellular staining, cells were permeabilized and fixed using the FOXP3 staining buffer kit (ThermoFisher Scientific, Waltham, MA, 00-5523-00) according to the manufacturer's instructions.

To detect apoptotic rates of DCs, washed cells were resuspended in 100 μL binding buffer and incubated with Annexin-V-PE (1:20; BD Biosciences, 559763, San Diego, CA) and 7-AAD (1:20; BD Biosciences, 559763, San Diego, CA) for 15 min at room temperature. Flow cytometry analyses were performed on an LSR II instrument (BD Biosciences, Mountain View, CA). Results were exported from FACSDiva V 7.0 software (BD Biosciences, Mountain View, CA) and analyzed using FlowJo Version 10.0 software.

***Western blot***

Cells were collected and lysed using RIPA lysis buffer (Huaxingbio, HX1862-1, Beijing, China). Lysates were incubated on ice for 30 min, then centrifuged at 12,000 rpm for 15 min at 4°C. The supernatants were denatured at 95°C for 5 min in the presence of 1× sodium dodecyl sulfate (SDS) loading buffer. Protein concentrations were measured using a BCA kit. Equal amounts of protein samples were loaded onto and separated by 8%-12% SDS polyacrylamide gel electrophoresis (Jingcai Biotechnology Co., PE008, Xi’an, China), then transferred to polyvinylidene fluoride membranes. The membranes were incubated with primary antibodies followed by HRP-conjugated anti-mouse or anti-rabbit secondary antibodies.

The primary antibodies used were: RETREG1 (1:1000; Abcam, ab151755, Cambridge, MA), RETREG1 (1:1000, Proteintech, 21537-1-AP, Wuhan, China), Phospho-RETREG1 (Ser-151) (1:1000, Wenyuange Biotech, Shanghai, China), HSPA5 (1:1000; Abcam, ab21685, Cambridge, MA), ATF4 (1:1000; Abcam, ab184909, Cambridge, MA), ATF6 (1:1000; Abcam, ab37149, Cambridge, MA), MAP1LC3B (1:1000; Cell Signaling Technology, 83506, Danvers, MA), SEC61B (1:1000; Cell Signaling Technology, 14648, Danvers, MA), EIF2AK3 (1:1000; Cell Signaling Technology, 5683, Danvers, MA), Phospho-EIF2AK3 (1:1000; Cell Signaling Technology, 3179, Danvers, MA), EIF2A (1:1000; Cell Signaling Technology, 5324, Danvers, MA), Phospho-EIF2A (1:1000; Cell Signaling Technology, 3398, Danvers, MA), and DDIT3 (1:1000; Cell Signaling Technology, 2895, Danvers, MA), ERN1 (1:1000; Cell Signaling Technology, 3294, Danvers, MA), XBP1 (1:1000; Cell Signaling Technology, 40435, Danvers, MA), Histone3 (1:1000; Abcam, ab1791, Cambridge, MA), MARCH 8 (1:1000, Absin, abs143503, Shanghai, China), MARCH1 (1:1000, Absin, abs139001, Shanghai, China), CCGP1 (1:1000, Proteintech, 13861-1-AP, Wuhan, China), CALCOCO1 (1:1000, Proteintech,19843-1-AP, Wuhan, China), ATL3 (1:1000, Proteintech, 16921-1-AP, Wuhan, China), TRIM13 (1:100, [Santa Cruz Biotechnology](https://www.baidu.com/link?url=tQTHuXU3BiiS5iJQO0KK7R9EPZPCjlEM5nY6S2J4u4q&wd=&eqid=f480ae3600ace3870000000366924c86), sc-398129, Dallas, TX), SEC62 (1:1000; Abcam, ab140644, Cambridge, MA), TEX264 (1:1000, Proteintech, 25858-1-AP, Wuhan, China), RTN3 (1:1000; Proteintech, 12055-2-AP, Wuhan, China), BAX (1:1000; Cell Signaling Technology, 2772, Danvers, MA), BCL-2 (1:1000; Cell Signaling Technology, 3498, Danvers, MA), Cleaved Caspase3 (1:1000; Cell Signaling Technology, 9664, Danvers, MA). β-actin was used as a loading control. Blots were visualized and analyzed using the ECL system to determine the relative expression levels of the specified proteins. Immunoblots were cropped from different parts of the same gel, and brightness or contrast were adjusted globally to enhance clarity.

***Label-free quantitative proteomics analysis***

Samples were sonicated three times on ice using a high-intensity ultrasonic processor (Scientz) in lysis buffer (8 M urea, 1% protease inhibitor cocktail). Debris was removed by centrifugation at 12,000 g for 10 min at 4°C. The supernatant was collected, and protein concentration was determined using a BCA kit. For digestion, the protein solution was reduced with 5 mM dithiothreitol for 30 min at 56°C, then alkylated with 11 mM iodoacetamide for 15 min at room temperature in the dark. The protein sample was diluted with 100 mM TEAB to reduce the urea concentration to less than 2 M. Trypsin was added at a 1:50 trypsin-to-protein mass ratio for the first overnight digestion, followed by a second digestion at a 1:100 trypsin-to-protein mass ratio for 4 hours. Peptides were desalted using a C18 SPE column.

For Liquid Chromatography-Mass Spectrometry (LC-MS) analysis, tryptic peptides were dissolved in solvent A (0.1% formic acid, 2% acetonitrile in water) and loaded directly onto a home-made reversed-phase analytical column. Peptides were separated using solvent B (0.1% formic acid in 90% acetonitrile) over 60 min, with a gradient of 25% to 35% in 22 minutes, increasing to 80% in 4 minutes, and held at 80% for the final 4 minutes, all at a constant flow rate of 450 nL/min on an EASY-nLC 1200 UPLC system (ThermoFisher Scientific, Waltham, MA). The separated peptides were analyzed on a Q Exactive™ HF-X (ThermoFisher Scientific, Waltham, MA) with a nano-electrospray ion source. The electrospray voltage was set to 2.0 kV. Full MS scan resolution was set to 60,000 for a scan range of 350-1600 m/z. Up to the 20 most abundant precursors were selected for further MS/MS analysis with a 30-second dynamic exclusion. HCD fragmentation was performed at a normalized collision energy (NCE) of 28%. Fragments were detected in the Orbitrap at a resolution of 30,000. MS/MS data were processed using the MaxQuant search engine (v.1.6.15.0). Tandem mass spectra were searched against the human SwissProt database concatenated with a reverse decoy database. The mass tolerance for precursor ions was set to 20 ppm in the first search and 5 ppm in the main search, and the mass tolerance for fragment ions was set to 0.02 Da. The false discovery rate (FDR) was adjusted to < 1%. GO and KEGG pathway enrichment analyses were performed based on DEPs.

***Immunoprecipitation and ubiquitination assay***

For the MHC-II ubiquitination assay, DCs were treated with 10 μM MG132 prior to harvest. Cells were lysed in RIPA buffer supplemented with a protease inhibitor cocktail (Sigma-Aldrich, P834, St. Louis, MO). After pre-clearing with protein-A/G agarose beads (ThermoFisher Scientific, 78610, Waltham, MA), the cell lysates were immunoprecipitated overnight at 4°C with an MHC-II antibody (1:1000; Invitrogen, 14-5321-82, Carlsbad, CA) or IgG control. The beads were then washed five times with lysis buffer, and the immunoprecipitates were subjected to immunoblot analysis using a ubiquitination antibody (1:1000; Santa Cruz Biotechnology, sc-8017, Dallas, TX).

***Co-******immunoprecipitation (Co-IP)*** ***assay***

Collected DCs were washed three times with PBS, and the supernatant was discarded. Cells were then lysed by adding an appropriate amount of pre-configured working solution (IP Lysis Buffer, phenylmethanesulfonyl fluoride [PMSF], and protein phosphatase inhibitor), followed by incubation on ice for 1 hour and three freeze-thaw cycles in liquid nitrogen. Cell lysates were centrifuged at 12,000 g for 30 min at 4°C, and the supernatant was collected for protein quantification. The primary antibody was added to the supernatants from the IP group, while an IgG antibody was added to the IgG group, and samples were incubated overnight at 4°C. Subsequently, IP and IgG samples were mixed with an appropriate amount of Protein A magnetic beads and incubated at 4°C with continuous rotation. The magnetic beads were then separated using a magnetic rack and washed five times with Wash Buffer. Precipitated proteins were eluted by boiling in SDS sample buffer for 10 minutes. The collected supernatants containing target proteins were analyzed using western blot analysis.

***Confocal microscopy***

The co-localization between the ER and autophagosomes or lysosomes, as well as morphological alterations of the ER, were assessed using laser scanning confocal microscopy (LSCM). Collected cells were fixed with 4% paraformaldehyde for 20 min at 4°C after three PBS washes, followed by permeabilization with blocking buffer (0.3% Triton X-100 in PBS; Sigma-Aldrich, T8787, St. Louis, MO) for 20 min at room temperature. After three more PBS washes, cells were blocked with 1% BSA (Sigma-Aldrich, A7030, B2064, St. Louis, MO) for 1 h at room temperature.

Subsequently, cells were incubated with primary antibodies, including RETREG1(1:200, Cell Signaling Technology, 83414, Danvers, MA), MARCH 8 (1:1000, Absin, abs143503, Shanghai, China), LAMP1 (1:100; Abcam, ab25630, Cambridge, MA) and CD11c (1:100, Santa Cruz Biotechnology, sc-398708, Dallas, TX) in 1% BSA overnight at 4°C. Following three PBS washes, cells were incubated with secondary antibodies [1:200, Anti-rabbit IgG (H+L), F(ab')_2_ Fragment (Alexa Fluor^®^ 488 Conjugate, Cell Signaling Technology, 4412, Danvers, MA] or [1:200, Anti-mouse IgG (H+L), F(ab')_2_ Fragment (Alexa Fluor^®^ 647 Conjugate), Cell Signaling Technology, 4410, Danvers, MA] for 1 h at room temperature. Cells were then stained with LC3B (1:50, Cell Signaling Technology, 18577, Danvers, MA), LysoTracker Deep Red (1:1000; Invitrogen, L12492, Carlsbad, CA), ER-Tracker Red (1:1000; Invitrogen, E34250, Carlsbad, CA) or ER-Tracker Green (1:1000; Invitrogen, E34251, Carlsbad, CA) or ER-Tracker Blue-White DPX (1:1000; Invitrogen, E12353, Carlsbad, CA) for 1 hour in a 5% CO_2_, 37°C incubator after three PBS washes.

Finally, after another three PBS washes, cells were stained with 4’,6-diamidino-2-phenylindole (DAPI; Sigma-Aldrich, D9542, St. Louis, MO) and mounted onto slides. The slides were observed using a laser scanning confocal microscope (Leica, Mannheim, Germany).

***Transmission electron microscopy***

After centrifugation and removal of the supernatant, collected cells were fixed with 2.5% glutaraldehyde at 4°C, followed by refixation with 1% osmium tetroxide. The specimens were subjected to acetone gradient dehydration, Epon812 embedding, semi-thin sectioning for optical positioning, and ultra-thin sectioning. Ultrathin sections were simultaneously stained with 2% uranyl acetate and 0.3% lead citrate. Reticulophagy and ER morphology were examined using an H-7650 transmission electron microscope (Hitachi, Tokyo, Japan).

***Isolation of human PBMCs***

Whole-blood samples were collected in anticoagulated blood collection tubes containing EDTA and diluted with precooled PBS containing 0.5 μM EDTA. The cell suspensions were carefully layered onto an equal volume of Ficoll-Paque Human Peripheral Blood Lymphocyte Separation Solution and centrifuged at 800 g for 30 min. The interphase containing PBMCs was harvested, and red blood cells were lysed with lysis buffer for 5 min. PBMCs were then washed and resuspended in RPMI 1640 (Solarbio, 31800, Beijing, China) for subsequent experiments.

***Enrichment of circulating Pan-DCs***

Pan-DC enrichments were performed immediately after obtaining PBMCs. The selection of pan-DCs was carried out according to the instructions of the EasySep Human Pan-DC Enrichment Kit (StemCell Technologies, 19251, Vancouver, CA). Briefly, PBMCs were adjusted to a concentration of 5×10^⁷^ cells/mL using PBS containing 2% FBS and 1 mM EDTA. Next, 30 μL/mL FcR Blocker, 50 μL/mL Pan-DC Enrichment Cocktail Component A, and 50 μL/mL Pan-DC Enrichment Cocktail Component B were added to each sample, followed by incubation at room temperature for 30 min. Thereafter, 100 μL/mL Magnetic Particles were added to the sample and incubated at room temperature for 10 min. After mixing and adjusting to the appropriate volume, the tube containing the cell suspensions was placed into a magnet and incubated for 5 min. The enriched pan-DC suspension was then transferred to a new tube for subsequent experiments.

***Analysis of public scRNA-seq datasets***

Publicly available scRNA-seq datasets containing CD45^+^ cells and DCs from PBMCs of patients were downloaded from the Single Cell Portal (SCP, https://singlecell.broadinstitute.org/) using accession code SCP548. scRNA-seq analyses were performed on enriched circulating DCs in the dataset using the ‘Seurat’ package in R software. DCs with fewer than 200 detected genes and mitochondrial content greater than 10% were removed during the quality control process. Filtered unique molecular identifiers (UMIs) were normalized using the ‘NormalizeData’ function.

Highly variable genes were identified using the ‘FindVariableGenes’ function with default parameters, followed by principal component analysis (PCA) based on these genes using the ‘RunPCA’ function. Bidimensional coordinates of single cells were obtained using the ‘RunUMAP’ function. For cell clustering analysis and visualization on a uniform manifold approximation and projection (UMAP) plot, the ‘FindClusters’ function was used based on the same principal components identified in the ‘RunUMAP’ function.

Differentially expressed genes (DEGs) across subclusters were identified using the ‘FindAllMarkers’ function on normalized data, with P-values adjusted using Bonferroni correction. Differential expression in each subpopulation was assessed using the Wilcoxon rank sum test in ‘Seurat’, and results were displayed as a heatmap. Upregulated DEGs in monocyte subtypes with P-values less than 0.05 and log2 fold change (FC) exceeding 0.5 were compared and visualized using volcano plots.

Pseudo-time trajectory analyses were performed using ‘Monocle2’ based on signature genes identified by the ‘DifferentialGeneTest’ function. Generalized additive models (GAMs) were constructed to generate the average expression of isoforms. Developmental trajectories among DC subsets were analyzed using ‘Monocle’ with default parameters.

**Supplementary Figures**


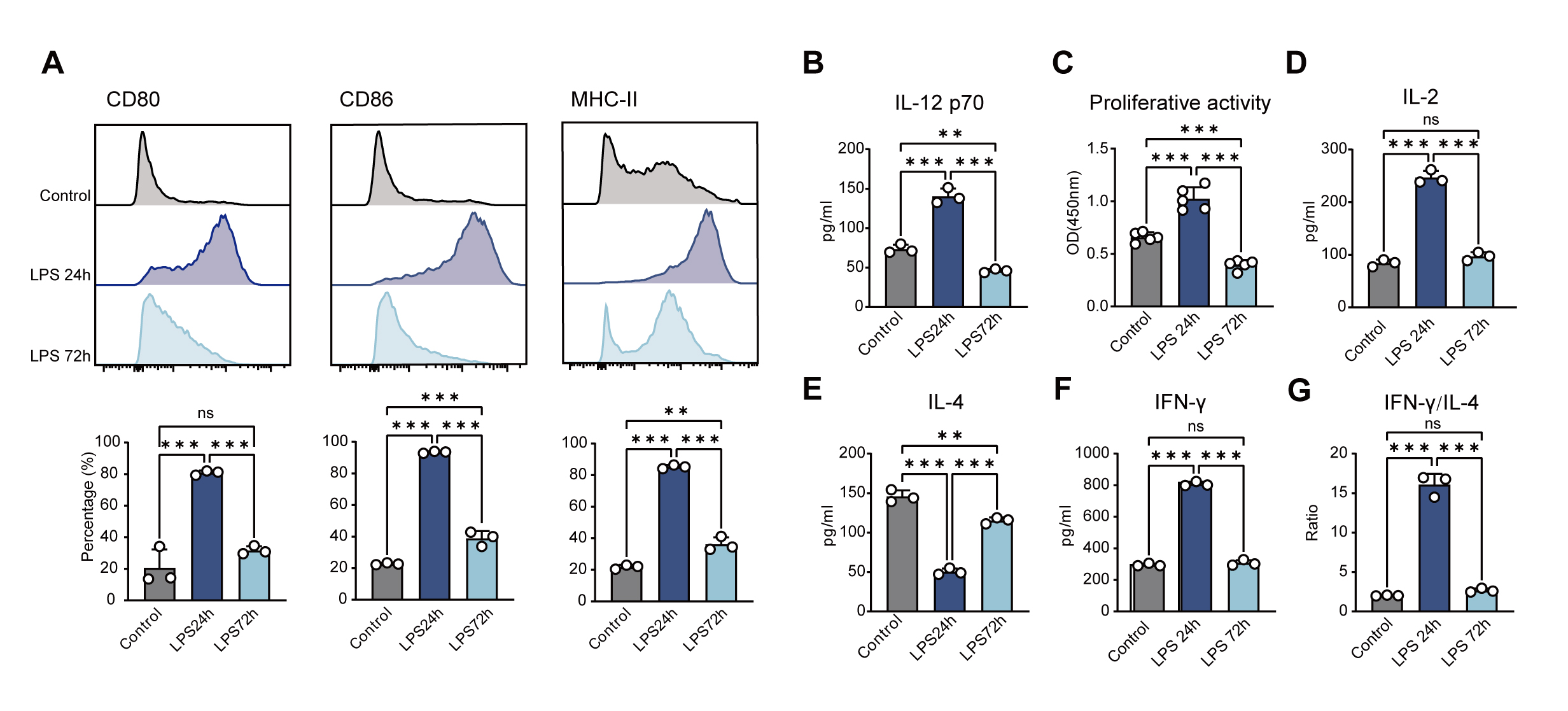


**Figure S1. Bacterial LPS drives DCs activation.** Splenic DCs derived from wild-type (WT) mice were stimulated with LPS (1 μg/mL) for 0, 24, and 72 h, followed by subsequent experiments. **A** Histogram and quantitative bar charts displaying the expression levels of CD80, CD86, and MHC-II, as measured by flow cytometry (*n* = 3). **B** ELISA analysis quantifying IL-12 p70 levels in culture supernatants (*n* = 3). **C** CCK8 assay measuring the proliferative activity of CD4^+^ T cells co-cultured with DCs (*n* = 5). **D-G** ELISA analysis of IL-2, IL-4, and IFN-γ levels in co-culture supernatants of panel C (*n* = 3).

Each sample in panels (**A-G**) was assayed in technical triplicate, with mean values representing that sample. Data in panels (**A-G**) represent mean ± SD. Statistical analysis was performed using one-way ANOVA followed by Tukey’s post hoc test. ns, not significant; ***P*<0.01, ****P*<0.001.


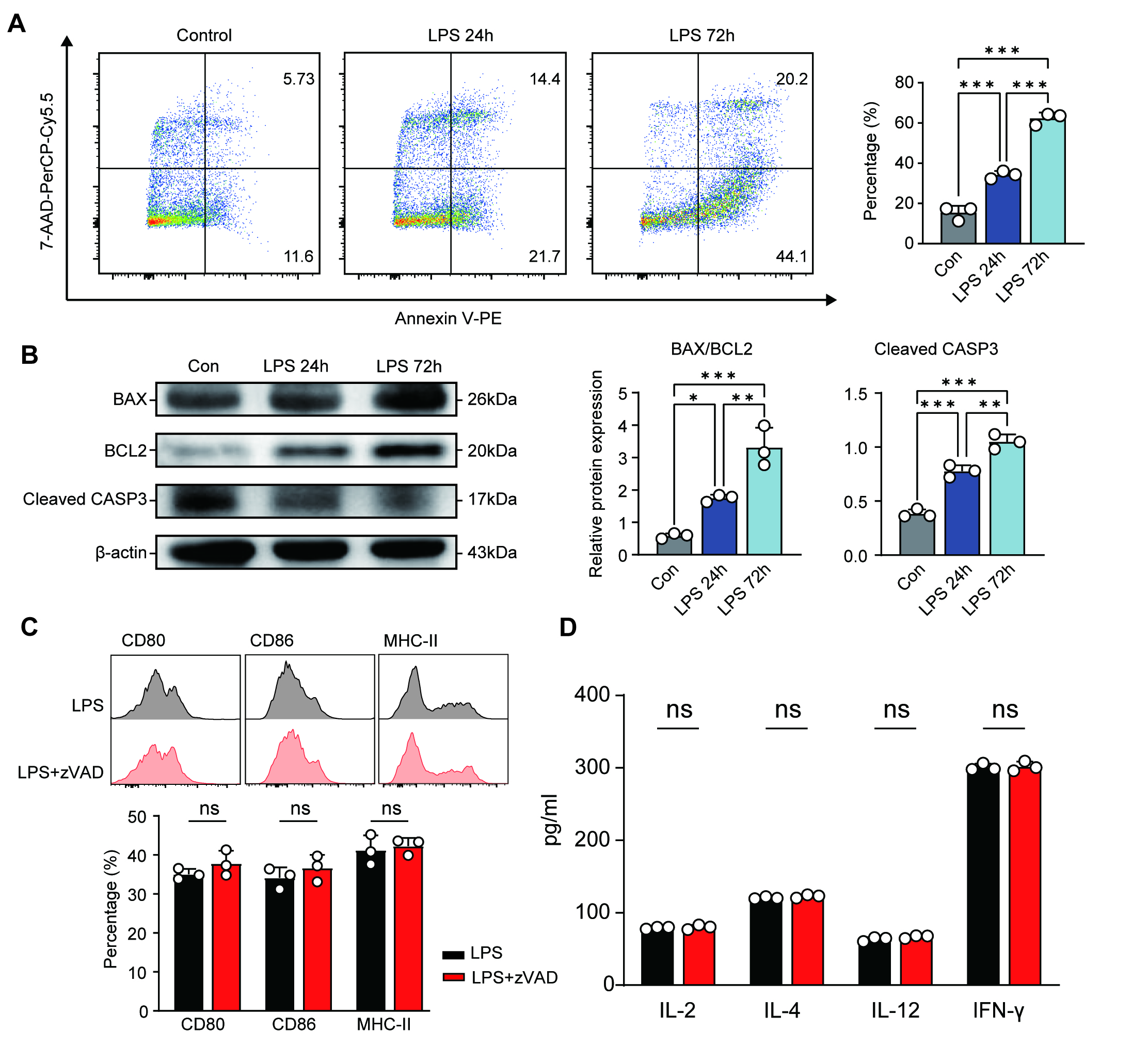


**Figure S2. Inhibiting DC apoptosis has no impact on functional deficit in late sepsis. A, B** Splenic DCs derived from WT mice were treated with LPS (1 μg/mL) for 0, 24, and 72 h, followed by evaluation of DCs apoptosis. **A** Representative flow cytometry plots with quantitative bar charts showing the apoptotic rate (UR + LR) of LPS-treated DCs (*n* = 3). **B** Western blot analysis of apoptosis-related proteins in LPS-treated DCs, including cleaved CASP3, BAX, and BCL2 (*n* = 3). **C, D** WT splenic DCs were treated with 1 μg/mL LPS for 72 h in the absence or presence of 20μM Z-VAD-FMK (zVAD, a pan-Caspase inhibitor). **C** Flow cytometry analysis of CD80, CD86, and MHC-II expression levels (*n* = 3). **D** ELISA assay of levels of IL-2, IL-4, IL-10, IFN-γ, and TGF-β in co-culture supernatants (*n* = 3).

Each sample in panels (**A, C, D**) was assayed in technical triplicate, with mean values representing that sample. For panel (**B**), each protein sample was loaded onto three independent gels, and mean densitometric values were used for inter-group comparisons. Data in panels (**A-D**) represent means ± SD. Statistical analysis was performed using one-way ANOVA followed by Tukey’s post hoc test for panels (**A, B**) or unpaired Student’s t-test for panels (**C, D**). ns, not significant; **P*<0.05, ***P*<0.01, ****P*<0.001.


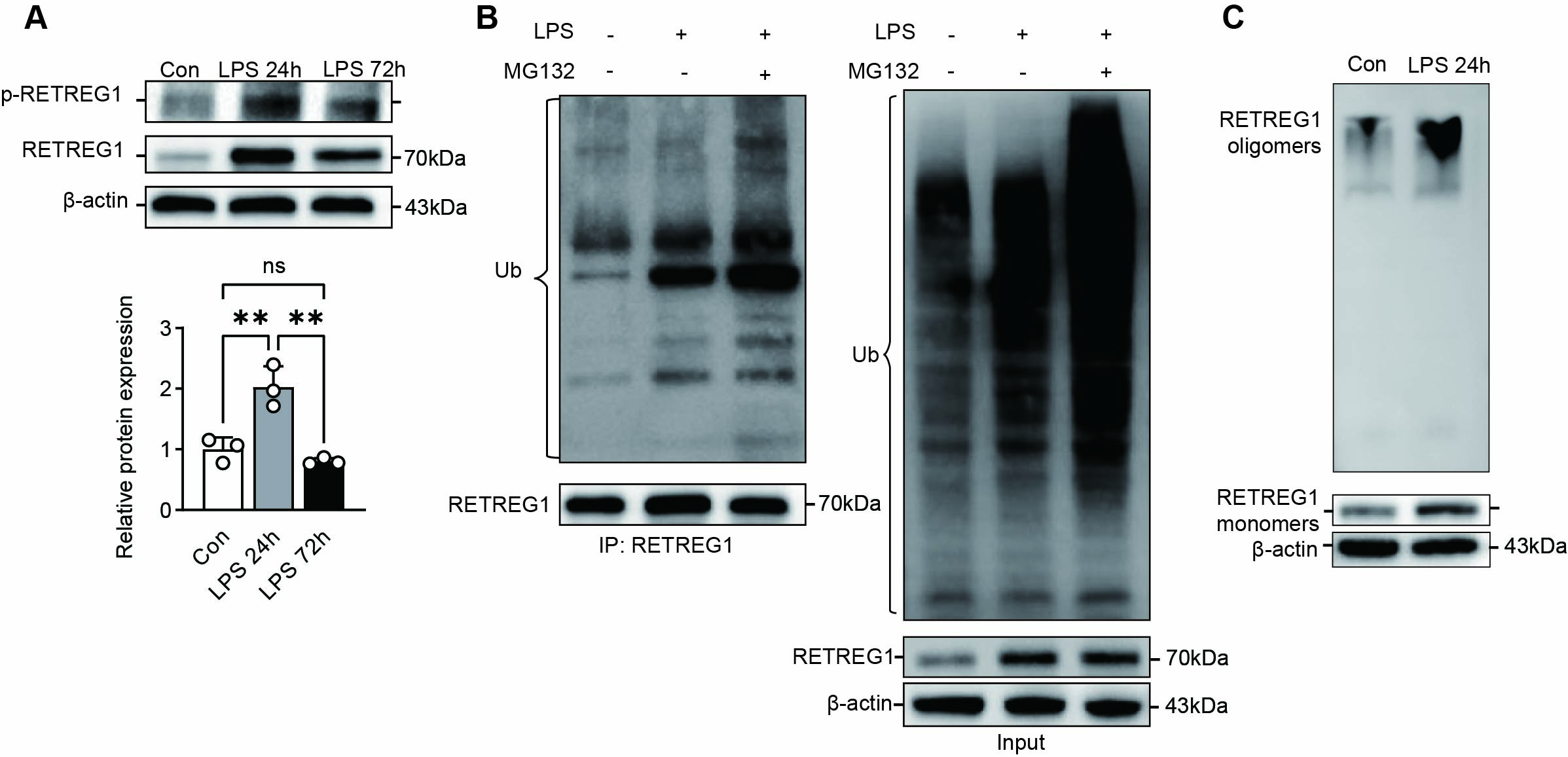


**Figure S3. Bacterial LPS facilitates posttranslational modifications and oligomerization of RETREG1 in DCs. A** Western blot analysis of the expression levels of p-RETREG1 in WT DCs primed with LPS (1 μg/mL) for 0, 24, and 72 h (*n* = 3). **B** WT DCs were primed with 1 μg/mL LPS for 24 h with the presence of 10 μM MG132, followed by immunoprecipitation analysis detecting the ubiquitinated level of RETREG1. **C** Western blot analysis showing the oligomer size of RETREG1 in DCs upon LPS stimulation.

Each protein sample in panel (**A**) was loaded onto three independent gels, and mean densitometric values were used for inter-group comparisons. Immunoblotting in panels (**B, C**) are representative of three independent experiments. Brightness and contrast adjustments were applied uniformly to the entire image to optimize presentation. Data in panel (**A**) represent means ± SD. Statistical analysis in panel (**A**) was performed using one-way ANOVA followed by Tukey’s post hoc test. ns, not significant; ***P*<0.01.


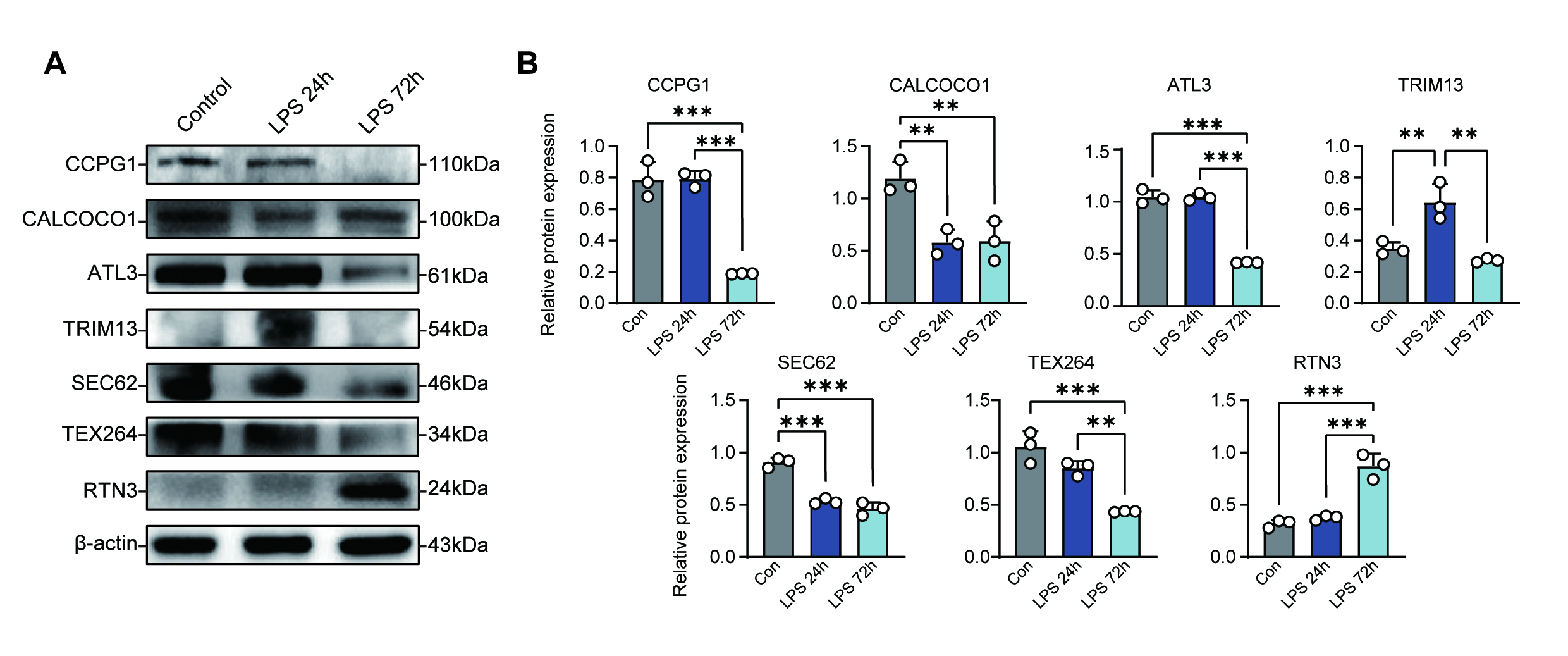


**Figure S4. Reticulophagic receptors exhibit distinct activation pattern upon LPS treatment. A** Splenic DCs derived from WT mice were treated with LPS (1 μg/mL) for 0, 24, and 72 hours, and the protein expression of reticulophagic receptors was assayed. **B** Relative levels of the indicated proteins were measured from 3 biologically independent samples (*n* = 3).

Each protein sample in panel (**B**) was loaded onto three independent gels, and mean densitometric values were used for inter-group comparisons. Data in panel (**B**) represent mean ± SD. Statistical analysis in panel (**B**) was performed using one-way analysis of variance (ANOVA) followed by Tukey’s post hoc test. ***P*<0.01 and ****P*<0.001.


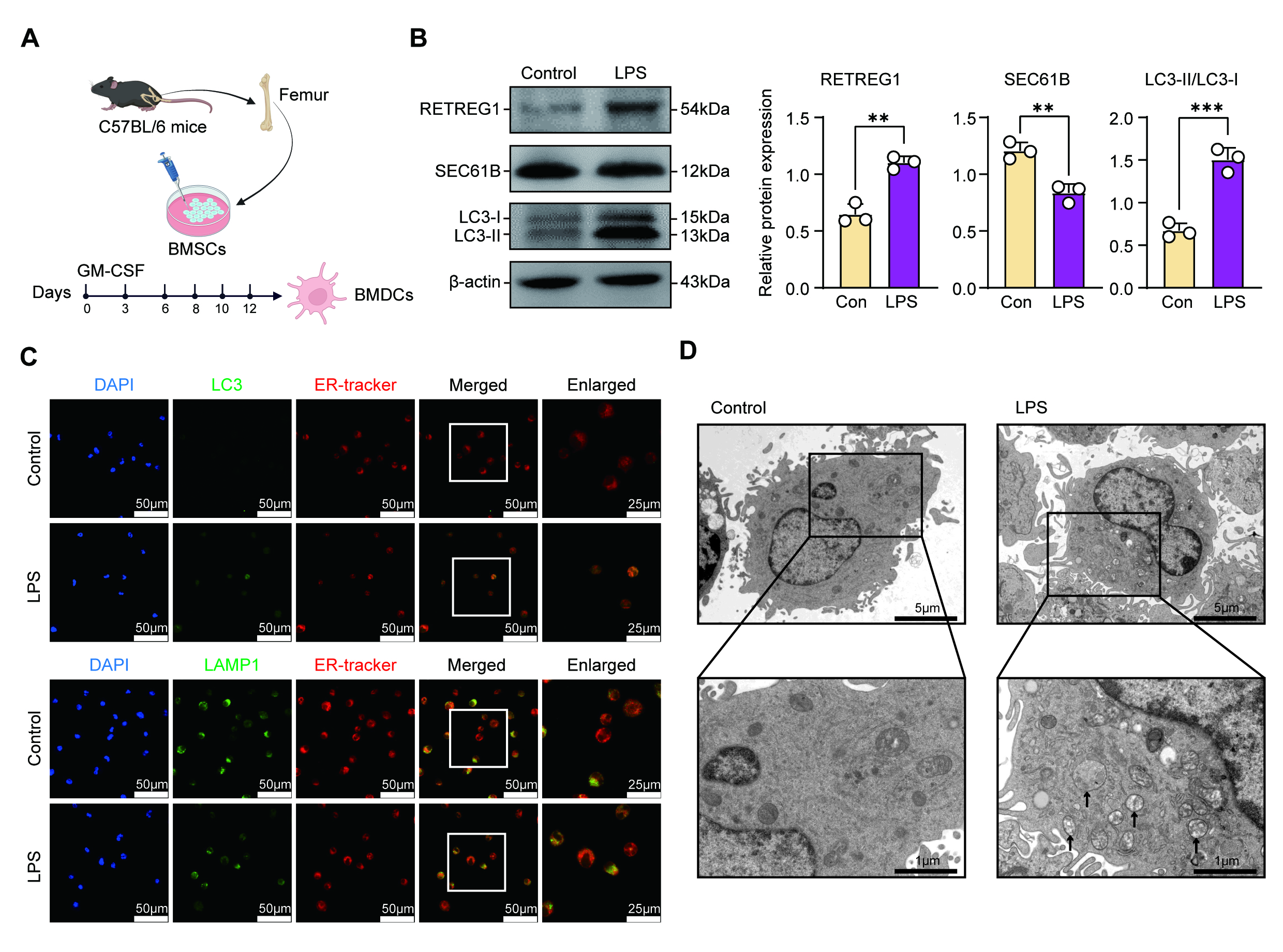


**Figure S5. LPS induces the activation of RETREG1-mediated reticulophagy in BMDCs.** **A** Schematic diagram depicting the process of isolating bone marrow cells from the femurs of WT mice and differentiating them into BMDCs using consecutive GM-CSF stimulation. Mature BMDCs were subsequently primed with LPS, followed by further experiments. **B** Western blot analysis of RETREG1, SEC61B, and LC3 in BMDCs following LPS (1 μg/mL) treatment for 24 h (*n* = 3). **C** Representative confocal immunofluorescence images showing ER colocalization with autophagosomes (upper panel) and lysosomes (lower panel) in BMDCs following LPS (1 μg/mL) treatment for 24 h. **D** TEM analysis illustrating the formation of ER-associated autophagosomes (arrows indicate ER within autophagosomes) in BMDCs following LPS (1 μg/mL) treatment for 24 h.

Protein sample in (**B**) was loaded onto three independent gels, and mean densitometric values were used for inter-group comparisons. Data in (**C, D**) are representative of three independent experiments. Data in panel (**B**) represent means ± SD. Statistical analysis in panel (**B**) was performed using an unpaired Student’s t-test. ***P*<0.01 and ****P*<0.001.


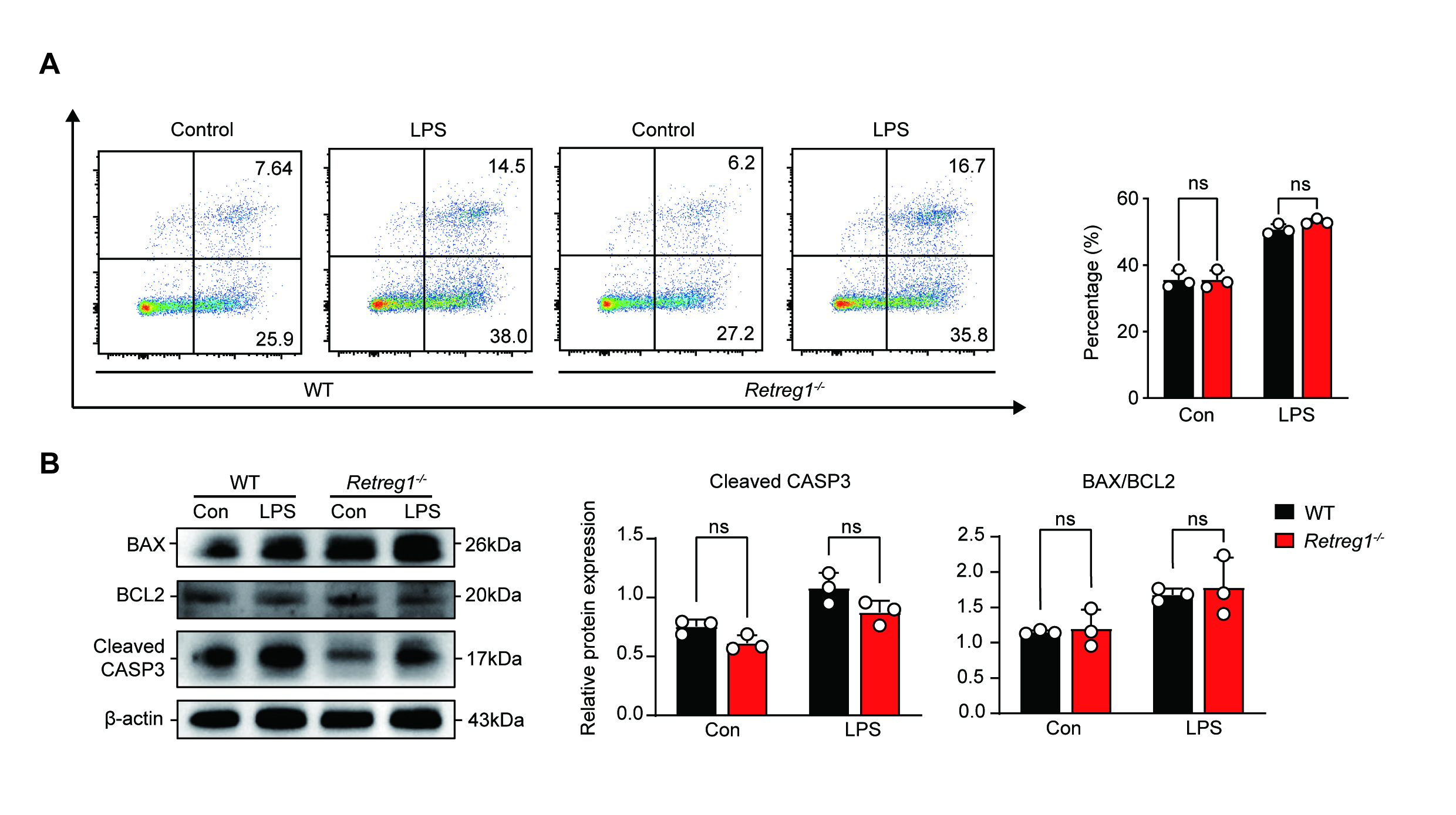


**Figure S6. Effect of** ***Retreg1* deficiency on LPS-induced apoptosis in DCs.** Splenic DCs derived from WT and *Retreg1^-/-^* mice were treated with LPS (1 μg/mL) for 24 h, followed by subsequent experiments. **A** Representative flow cytometry plots with quantitative bar charts showing the apoptotic rate (UR + LR) of DCs from each group (*n* = 3). **B** Western blot analysis of apoptosis-related proteins, including cleaved CASP3, BAX, and BCL2 (*n* = 3).

Each sample in (**A**) was assayed in technical triplicate, with mean values representing that sample. Protein sample in panel (**B**) was loaded onto three independent gels, and mean densitometric values were used for inter-group comparisons. Data in panels (**A, B**) represent means ± SD. Statistical analysis in panels (**A, B**) was performed using two-way ANOVA with Tukey’s post hoc test. ns, not significant.


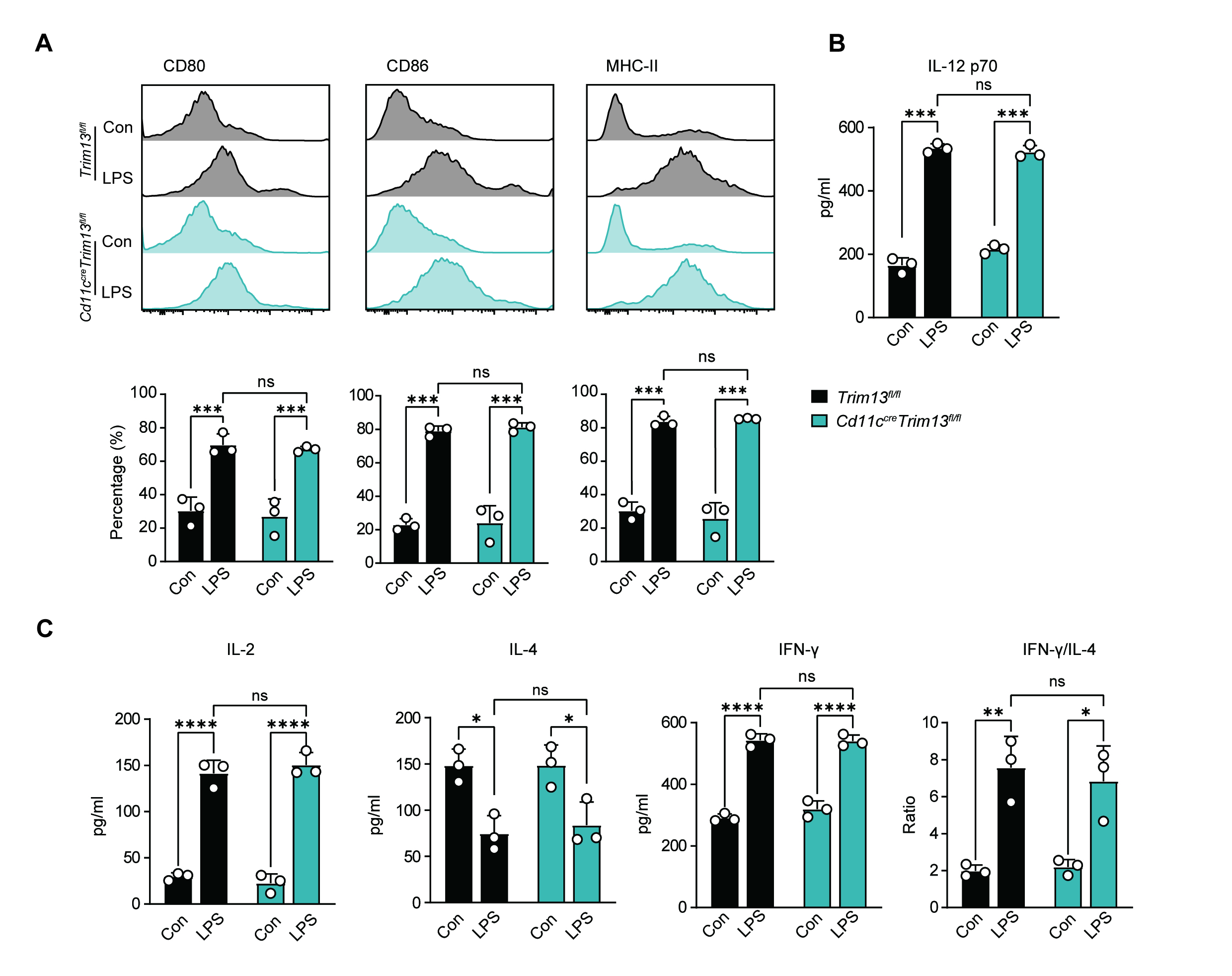


**Figure S7. TRIM13-dependent reticulophagy is not responsible for DC activation upon LPS challenge.** Splenic DCs obtained from *Trim13^flfl^* and *Cd11c^cre^Trim13^flfl^* mice were treated with LPS (1 μg/mL) or PBS for 24 h, followed by subsequent assays. **A** Flow cytometry analysis of CD80, CD86, and MHC-II expression levels (*n* = 3). **B** ELISA assay quantifying IL-12 p70 levels in culture supernatants (*n* = 3). **C** ELISA analysis of IL-2, IL-4, and IFN-γ levels in co-culture supernatants (*n* = 3).

Each sample in panels (**A-C**) was assayed in technical triplicate, with mean values representing that sample. Data in panels (**A-C**) represent means ± SD. Statistical analysis in panels (**A-C**) was performed using two-way ANOVA with Tukey’s post hoc test. ns, not significant; **P*<0.05, ***P*<0.01, ****P*<0.001.

**
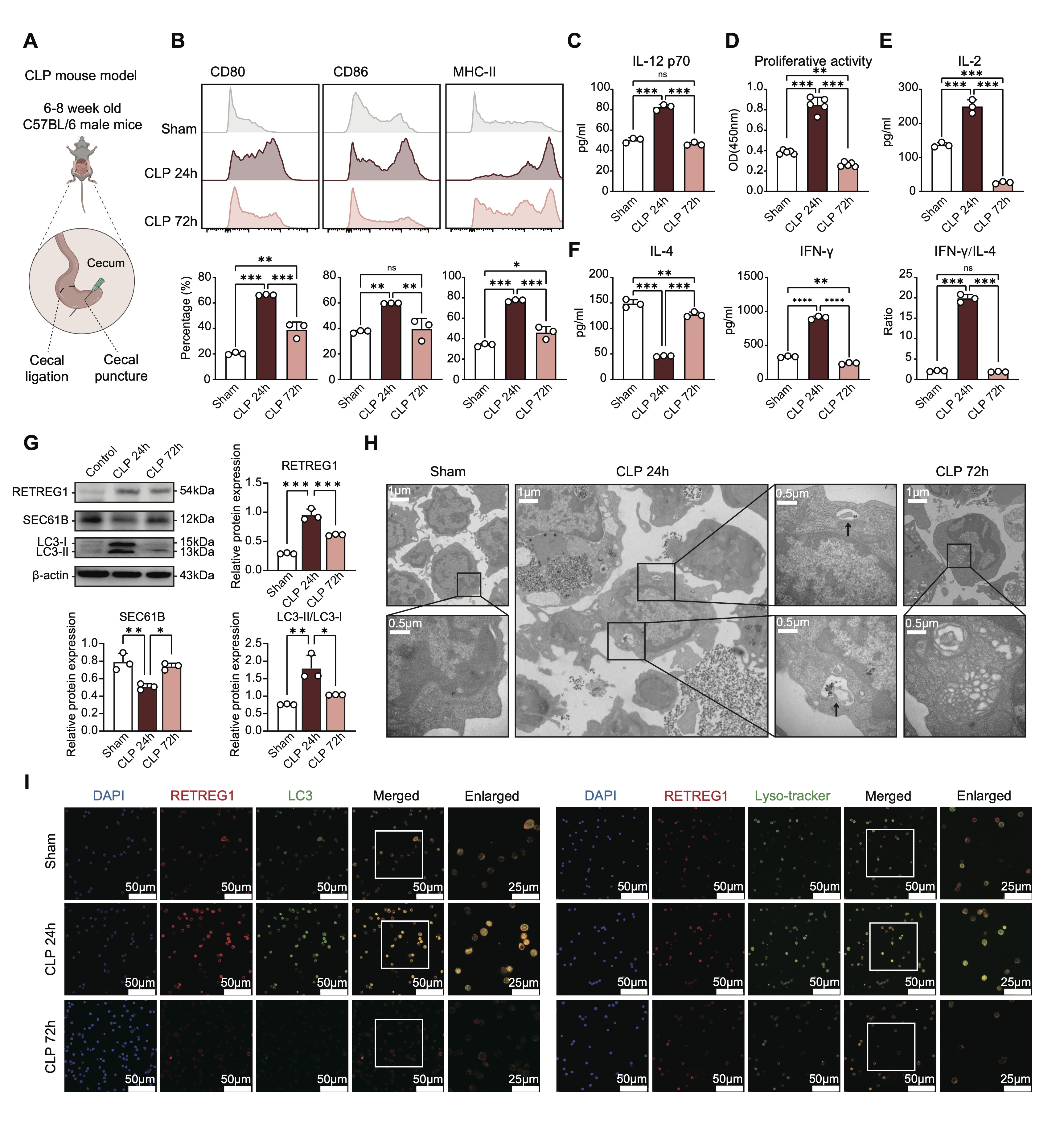
**

**Figure S8. Polymicrobial sepsis mobilizes RETREG1-dependent reticulophagy in DCs.** Splenic DCs were harvested at various intervals post-CLP surgery (0, 24, and 72 h) and subjected to subsequent experiments. **A** Schematic diagram depicting the experimental procedure of CLP surgery. **B** Histogram with quantitative bar charts showing the expression levels of CD80, CD86, and MHC-II (*n* = 3). **C** ELISA analysis of IL-12 p70 levels in culture supernatants (*n* = 3). **D** CCK8 analysis assessing the proliferative activity of CD4^+^ T cells (*n* =5). **E, F** ELISA analysis of IL-2, IL-4, and IFN-γ levels in co-culture supernatants (*n* = 3). **G** Western blot analysis of RETREG1, SEC61B, and LC3 protein expression (*n* = 3). **H** Representative TEM images showing the formation of ER-associated autophagosomes and ER morphology (arrows indicate ER within autophagosomes). **I** Representative immunofluorescence images showing RETREG1 colocalized with LC3 puncta (left panel) or LAMP1 (right panel).

Each sample in panels (**B-F**) was assayed in technical triplicate, with mean values representing that sample. For panel (**G**), each protein sample was loaded onto three independent gels, and mean densitometric values were used for inter-group comparisons. Data in panels (**H, I**) are representative of three independent experiments. Results in (**B-G**) are presented as means ± SD. Statistical analysis was performed using one-way ANOVA followed by Tukey’s post hoc test. **P*<0.05, ***P*<0.01, ****P*<0.001.


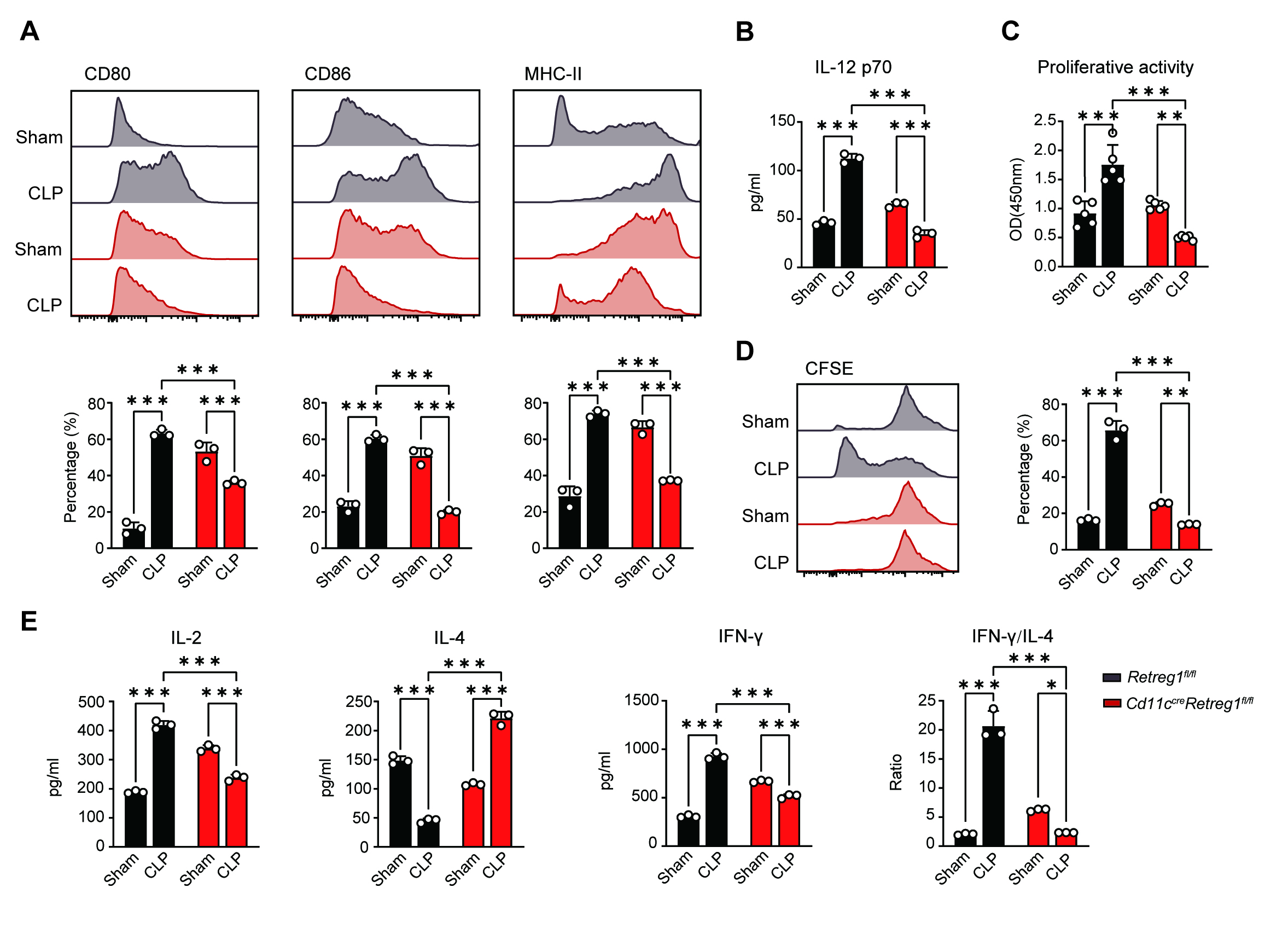


**Figure S9. RETREG1 mediates maturation and activation of DCs in polymicrobial sepsis.** Splenic DCs isolated from *Retreg1^fl/fl^* and *Cd11c^cre^Retreg1^fl/fl^* mice underwent CLP or sham surgery for 24 h were used for subsequent assays. **A** Flow cytometry analysis of functional molecules including CD80, CD86, and MHC-II (*n* = 3). **B** ELISA measurement of IL-12 p70 levels (*n* = 3). **C** CCK8 assay evaluating the proliferative activity of CD4^+^ T cells (*n* = 5). **D** Flow cytometry analysis determining the proportion of divided CD4^+^ T cells stained with CFSE. **E** ELISA analysis of IL-2, IL-4, and IFN-γ levels (*n* = 3).

For data in (**A-E**), each sample was assayed in technical triplicate, with mean values representing that sample. Data in panels (**A-E**) are presented as means ± SD. Statistical analysis in panels (**A-E**) was performed using two-way ANOVA with Tukey’s post hoc test. **P*<0.05, ***P*<0.01, ****P*<0.001.


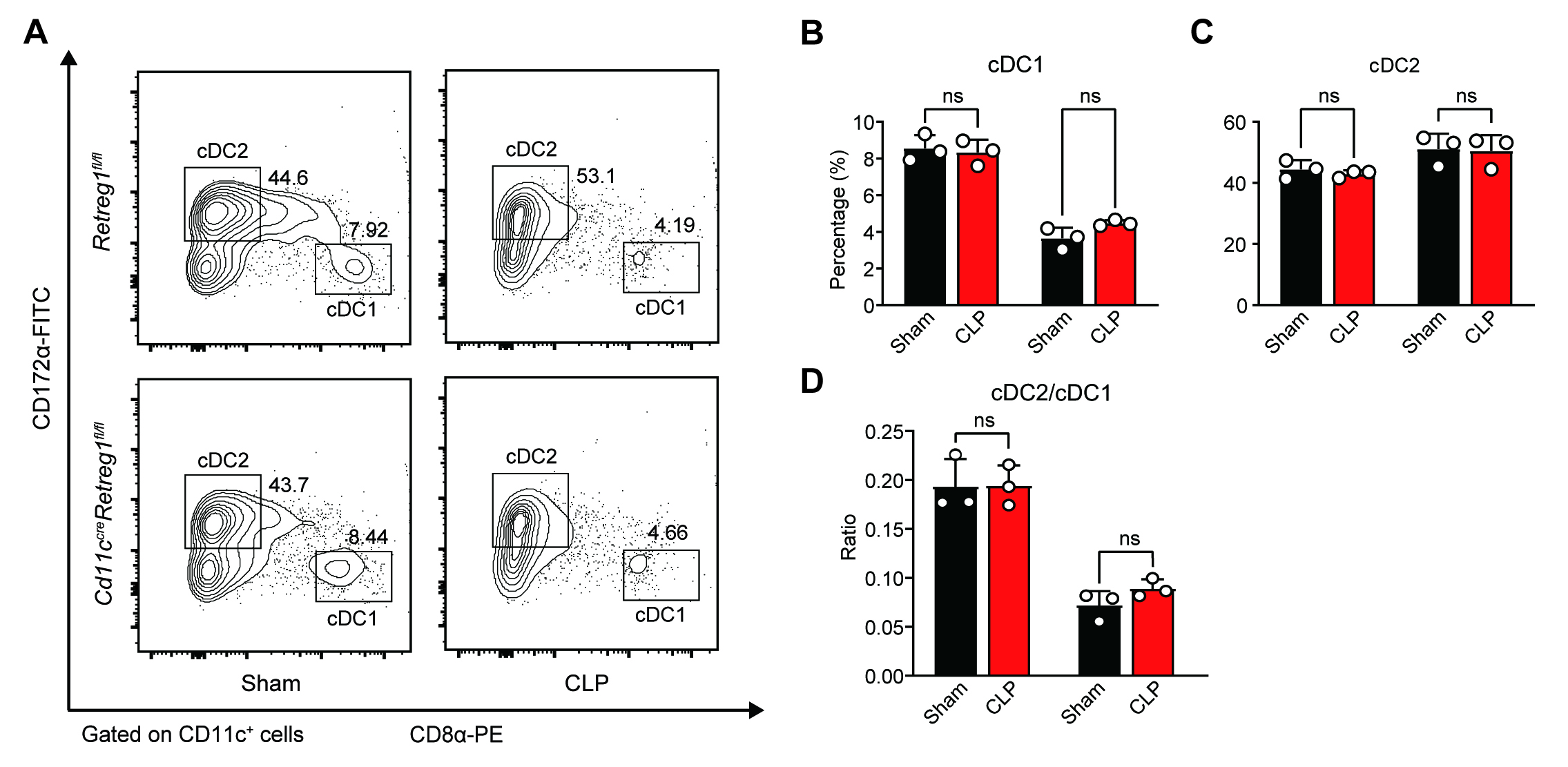


**Figure S10. RETREG1 was not responsible for DC differentiation in polymicrobial sepsis.** Splenic DCs isolated from *Retreg1^fl/fl^* and *Cd11c^cre^Retreg1^fl/fl^* mice that underwent CLP or sham surgery were processed into flow cytometry analysis. **A** Representative counter plot exhibiting proportion of cDC1 and cDC2 in CD11c^+^ cells from each group. **B** Bar plot showing the proportion of cDC1 in CD11c^+^ cells between groups (*n* = 3). **C** Quantitative bar plot comparing the proportion of cDC2 in CD11c^+^ cells between groups (*n* = 3). **D** Flow cytometry analysis of cDC2 to cDC1 ratio across groups (*n* = 3).

Each sample in panels (**B-D**) was assayed in technical triplicate, with mean values representing that sample. Data in panels (**A-E**) are presented as means ± SD. Statistical analysis in panels (**A-E**) was performed using two-way ANOVA with Tukey’s post hoc test. **P*<0.05, ***P*<0.01, ****P*<0.001.


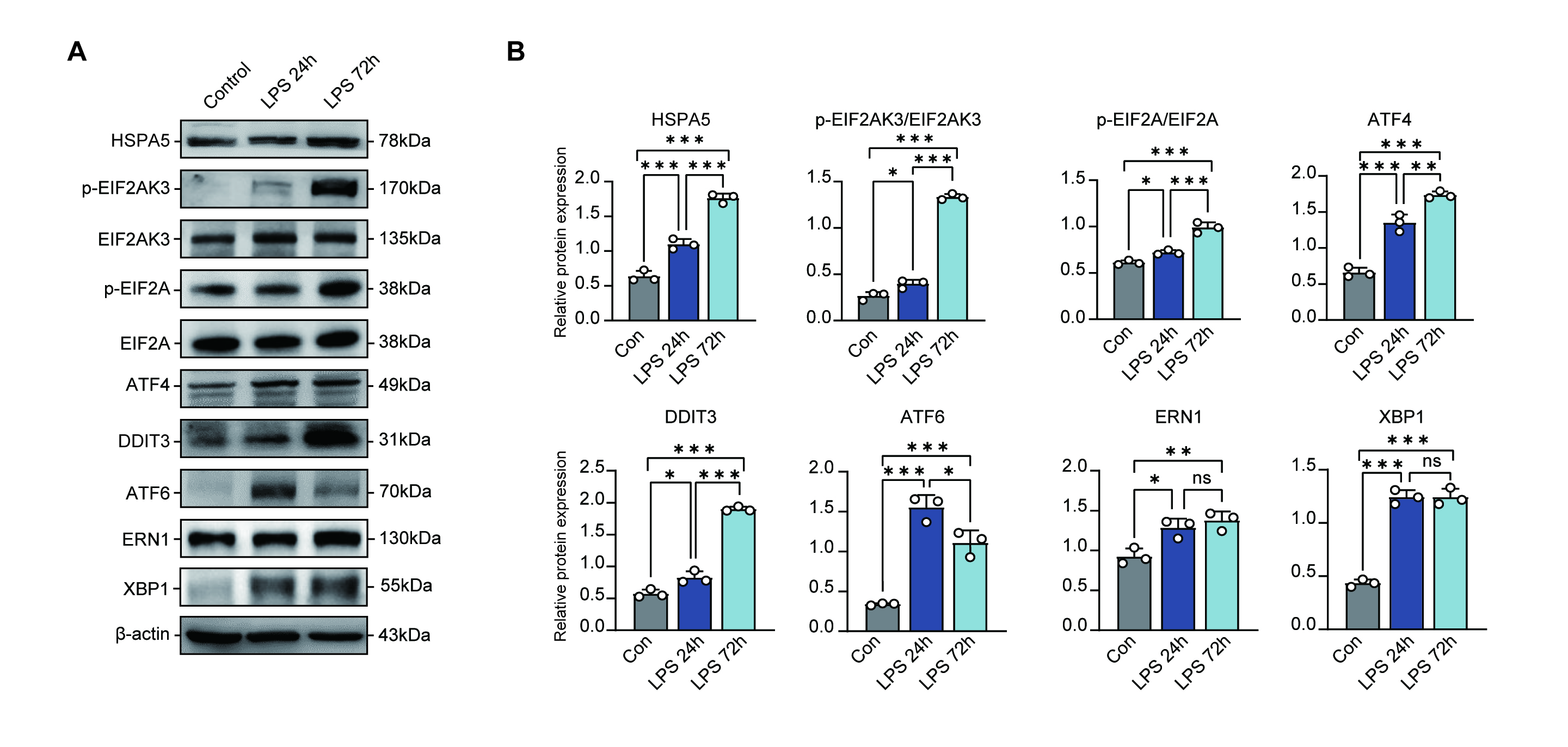


**Figure S11. Prolonged LPS exposure renders overactivation of ER stress in DCs. A** Western blot analysis of UPR-related proteins in splenic DCs following treatment with LPS (1 μg/mL for 0, 24, and 72 h). **B** Relative levels of the indicated proteins were measured from 3 biologically independent samples (*n* = 3).

Each protein sample in panel (**B**) was loaded onto three independent gels, and mean densitometric values were used for inter-group comparisons. Data in panels (**B**) represent mean ± SD. Statistical analysis in panels (**B**) was performed using one-way ANOVA and two-way ANOVA with Tukey’s post hoc test, respectively. ns, not significant; **P*<0.05, ***P*<0.01, ****P*<0.001.


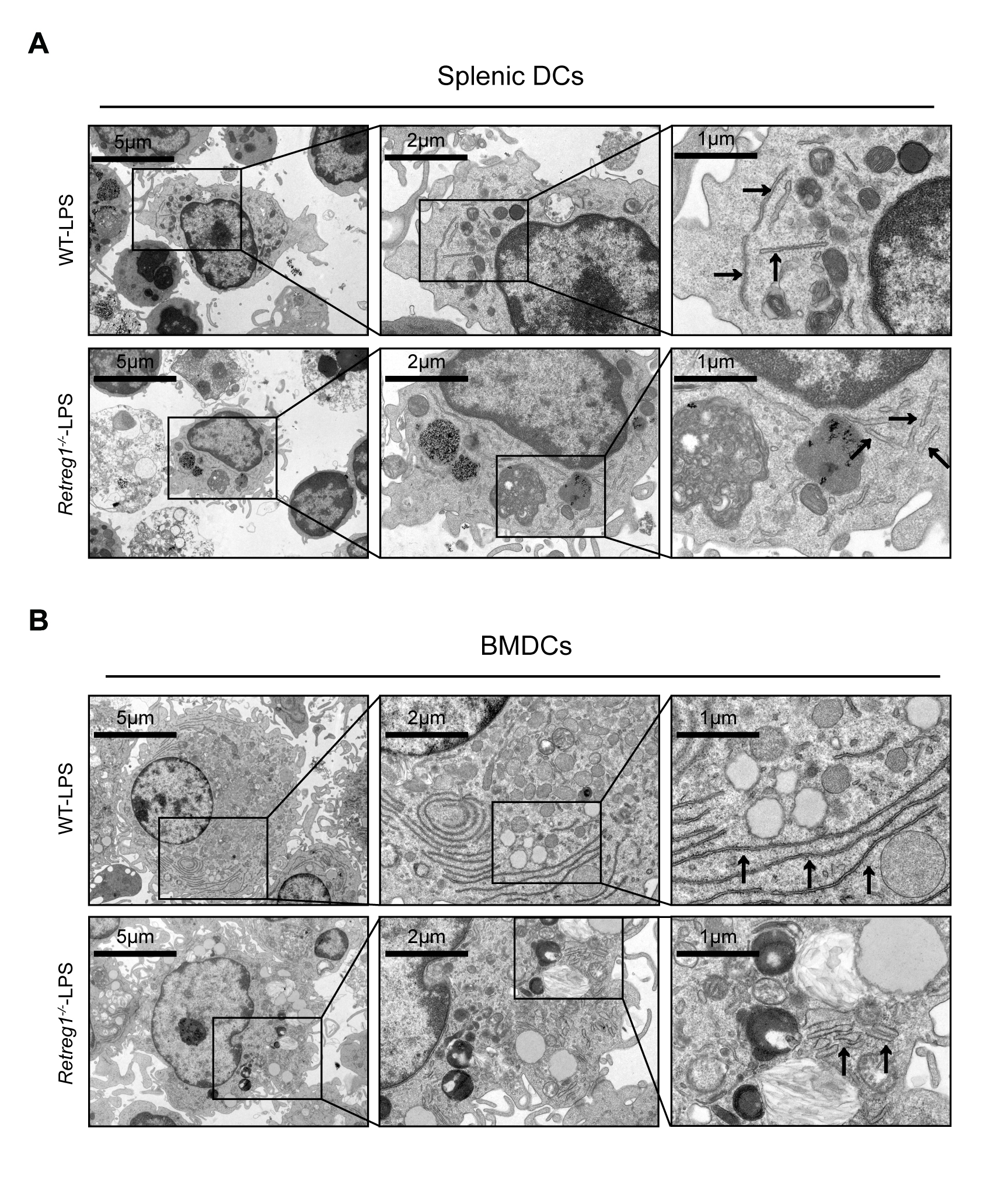


**Figure S12.** ***Retreg1* deficiency results in substantial expansion of ER membranes in LPS-challenged DCs. A** TEM analysis of ER morphology in splenic DCs collected from WT and *Retreg1*^-/-^ mice stimulated with 1 μg/mL LPS for 24 h. **B** Representative TEM images showing ER morphology in LPS-primed WT and *Retreg1*^-/-^ BMDCs. Images in (**A, B**) are representative of three independent experiments.


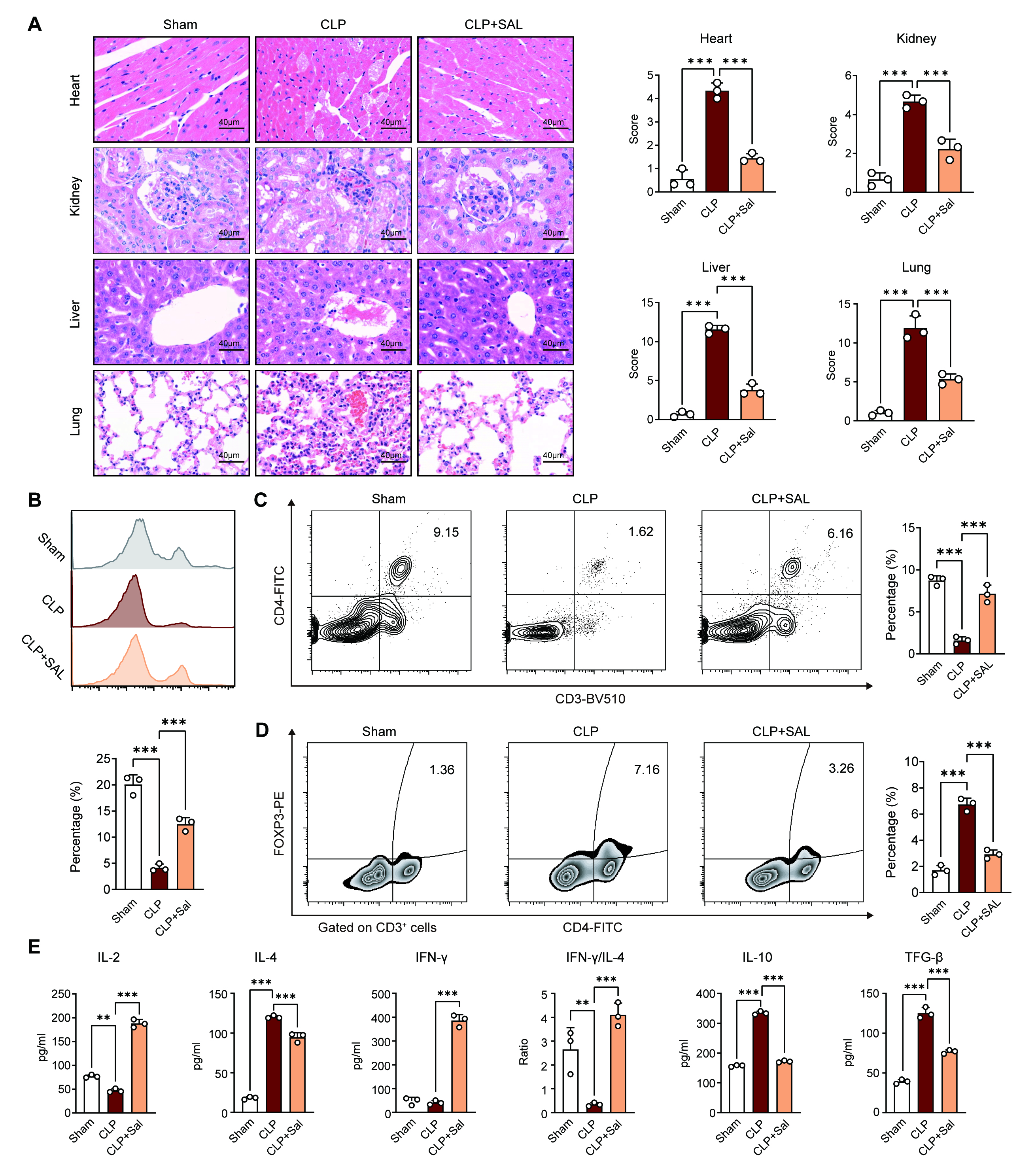


**Figure S13. Inhibiting EIF2A phosphorylation by salubrinal alleviates immune suppression and organ injuries caused by *Retreg1* deficiency.** Multiple organs**,** PBMCs and plasma were collected from *Cd11c^cre^Retreg1^fl/fl^* mice underwent sham or CLP surgery, with or without intraperitoneal injection of salubrinal (20 mg/kg, 1 h post CLP surgery). **A** Representative images (left panel) and histological scoring (right panel) of HE exhibiting the pathological alterations in mice (*n* = 3). **B** Histogram and quantitative bar charts indicating percentage of CD3^+^ cells in different groups (*n* = 3). **C** Counter plots with quantitative bar charts comparing proportion of CD3^+^CD4^+^ cells between groups (*n* = 3). **D** Representative counter plots with quantitative bar plots showing CD3^+^CD4^+^Foxp3^+^ Tregs proportion (*n* = 3). **E** ELISA analysis of circulating levels of IL-2, IL-4, IL-10, IFN-γ, TGF-β, and ratio of IFN-γ to IL-4 (*n* = 3).

Histological scoring in panel (**A**) five microscopic fields per specimen were evaluated and averaged. Each sample in panels (**B-E**) was measured in technical triplicate to minimize analytical bias. Results in panels (**A-E**) were presented as means ± SD. Statistics for panels (**A-E**) were calculated using one-way ANOVA followed by Tukey’s post hoc test. ***P* < 0.01 and ****P* < 0.001.


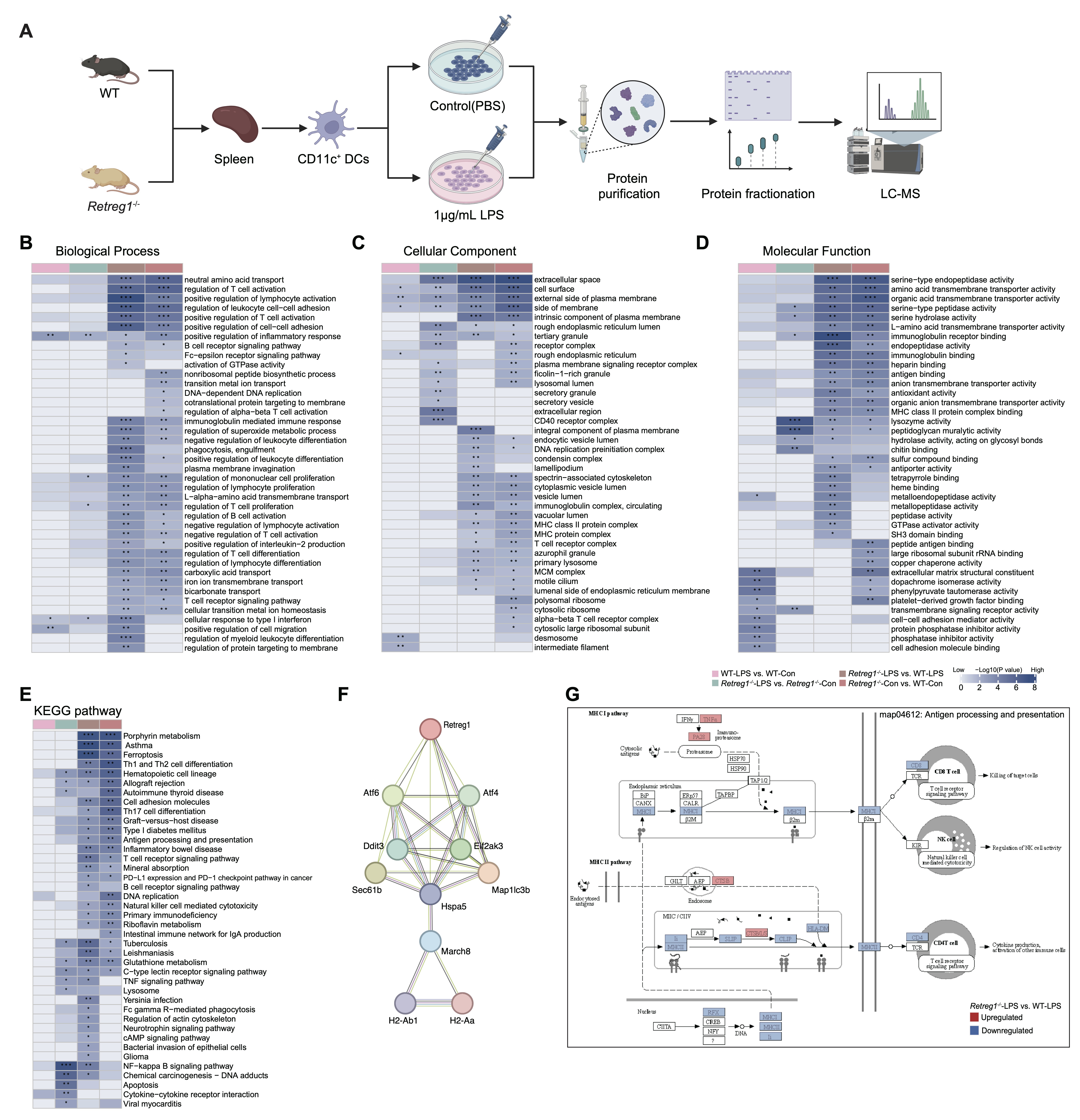


**Figure S14. Proteomic analysis identifies MHC-II as a key molecule affected by *Retreg1* deficiency.** LPS-treated (1 μg/mL) WT and *Retreg1*^-/-^ DCs were processed into mass spectrometry analysis. **A** Schematic workflow showing the experimental design of mass spectrometry analysis in the current study. **B-D** GO enrichment-based clustering analysis of DEPs across all comparisons. **E** KEGG pathway enrichment-based clustering of DEPs between groups. **F** PPI network analysis of DEPs between groups, conducted by using the STRING database (https://cn.string-db.org/). **G** Mapping of identified DEGs in antigen processing and presentation (KEGG PATHWAY Database [https://www.kegg.jp/kegg/pathway.html], map04612).

Statistical transformations in panels (**B-E**) were performed using the function x = −log10 (*P* value). These *P* values were then clustered by one-way hierarchical clustering in Genesis. **P*<0.05, ***P*<0.01, ****P*<0.001.

**
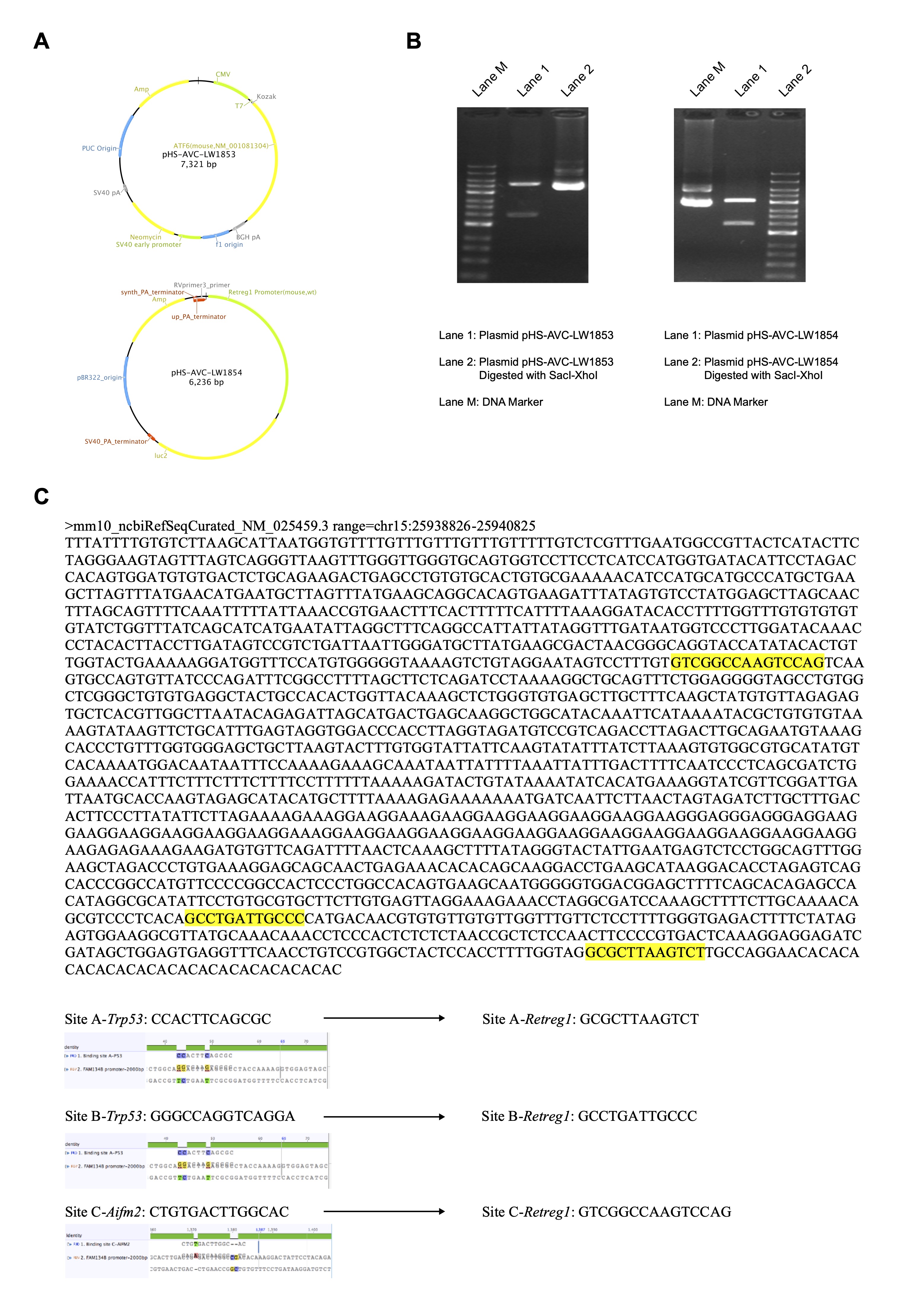
**

**Figure S15. Double luciferase reporter gene assay evaluates the binding activity of *Atf6* to *Retreg1*.** **A** Schematic representation of plasmids expressing ATF6 (upper panel) and the RETREG1 promoter (lower panel). **B** Restriction digestion map verifying the synthesized plasmids expressing ATF6 (left panel) and the RETREG1 promoter (right panel). **C** Identification and prediction of ATF6 binding sites on the promoter region of RETREG1 based on published literature. Three putative binding sites were identified for subsequent deletion, including two sites for regulating *Trp53* transcription and one for *Aifm2*.


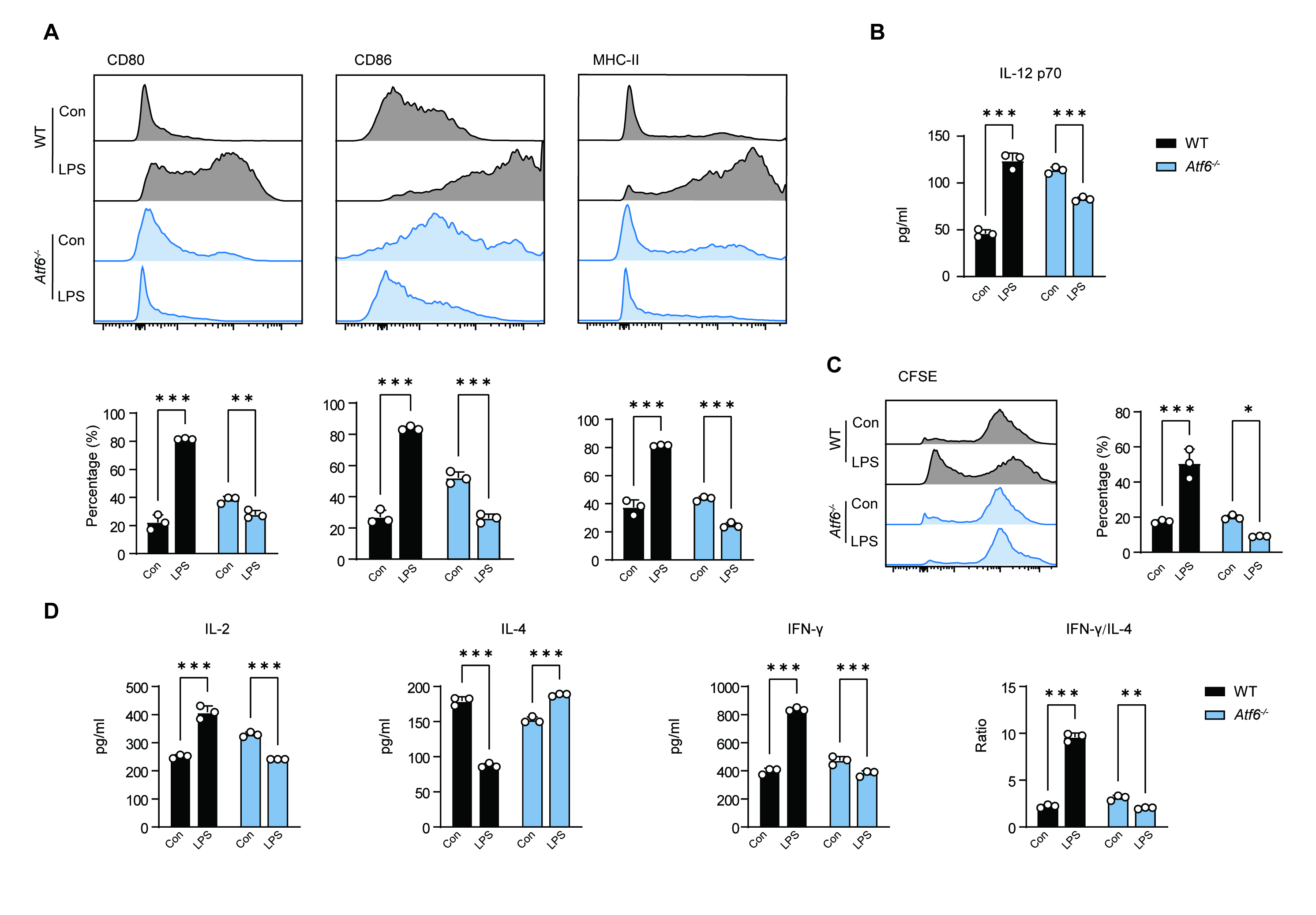


**Figure S16. *Atf6***^-/-^ **DCs exhibit defective functional phenotype with impaired priming activity for T cells.** **A-D** Splenic DCs harvested from WT mice and *Atf6*^-/-^ mice were treated with LPS (1 μg/mL) or PBS for 24 h followed by subsequent assays. **A** Flow cytometry analysis measuring expression levels of CD80, CD86, and MHC-II (*n* = 3). **B** ELISA analysis of levels of IL-12 p70 in cultural supernatants (*n* = 3). **C** Histogram with quantitative bar chart displaying proportion of divided CD4^+^ T cells stained with CFSE (*n* = 3). **D** ELISA measurement of IL-2, IL-4, and IFN-γ levels in co-cultural supernatants (*n* = 3).

For data in (**A-D**), each sample was assayed in technical triplicate, with mean values representing that sample. Data are merged from or representative of at least two independent experiments. Results in panels (**A-D**) were presented as means ± SD. Statistics in panels (**A-D**) were assessed by two-way ANOVA with Tukey’s post hoc test. ***P* < 0.01 and ****P* < 0.001.


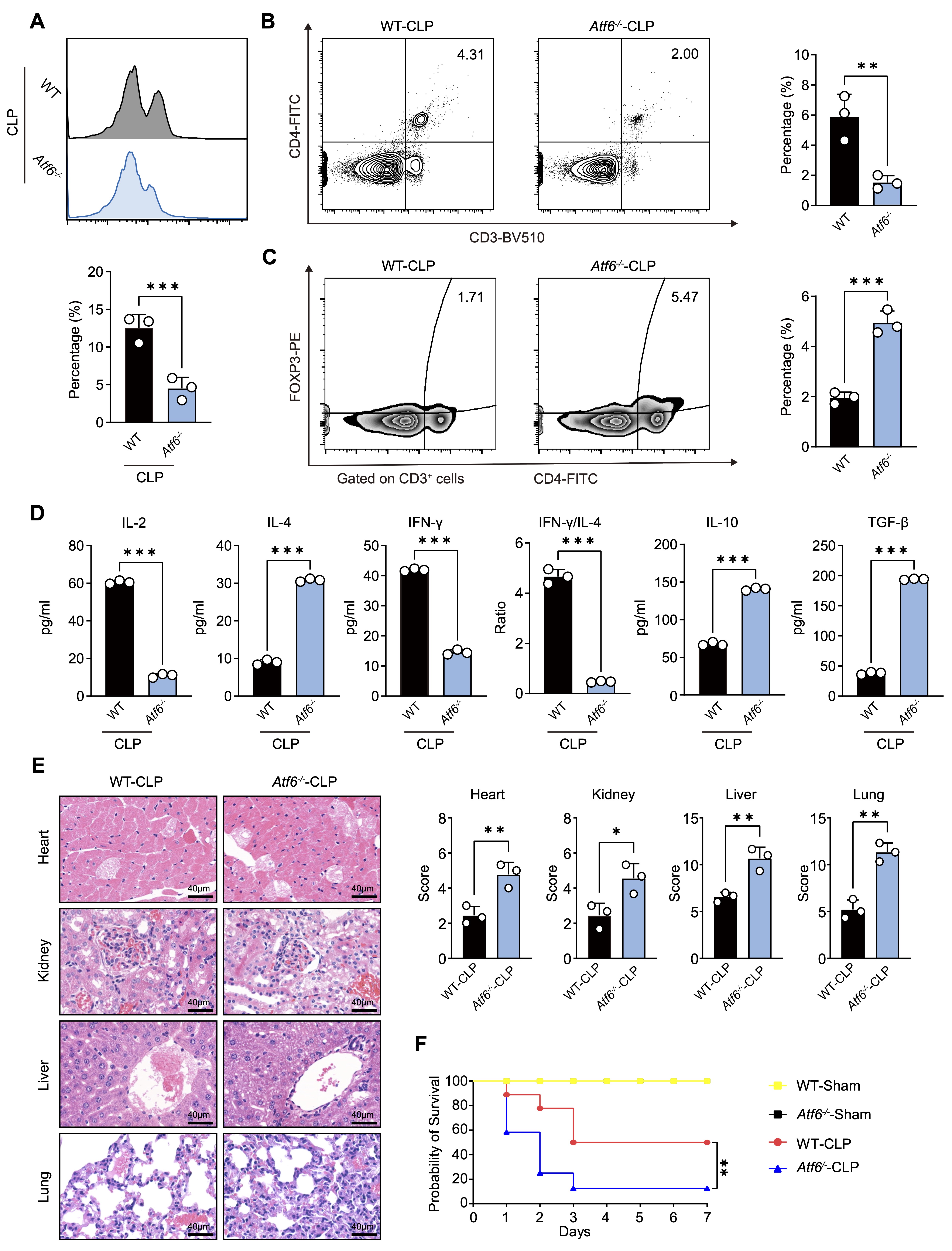


**Figure S17. *Atf6*** **deficiency aggregates immune suppression and organ lesions in CLP mice. A-D** PBMCs and plasma were obtained from WT and *Atf6*^-/-^ mice underwent CLP surgery, followed by subsequent assay. **A** Histogram with quantitative bar charts showing the proportion of CD3^+^ cells in CLP mice (*n* = 3). **B** Representative contour plots and quantitative bar charts exhibiting disparities in CD3^+^CD4^+^ T lymphocytes (*n* = 3). **C** Contour plots with quantitative bar charts comparing the percentage of Tregs in each group. **D** ELISA analysis examining serum levels of IL-2, IL-4, IL-10, IFN-γ, and TGF-β (*n* = 3). **E** Representative HE images (left panel) and histological scores (right panel) showing pathological changes in multiple organs harvested from septic mice (*n* = 3). **F** Survival analysis of WT and *Atf6^-/-^* mice within 7 days post-sham or CLP surgery (WT-Sham, *n* = 8; WT-CLP, *n* = 22; *Atf6^-/^*-Sham, *n* = 10; *Atf6^-/^*-CLP, *n* = 24).

Each sample in panels (**A-D**) was measured in technical triplicate to minimize analytical bias. Histological scoring in panel (**E**) five microscopic fields per specimen were evaluated and averaged. Data are in panel (**F**) are representative of three independent experiments. Data in panels (**A-D**) represent means ± SD. Statistical analysis for panels (**A-D**) was performed using one-way ANOVA followed by Tukey’s post hoc test. Statistical analysis in panel (**F**) was conducted using survival curve comparison with the log-rank test. **P*<0.05, ***P*<0.01, ****P*<0.001.


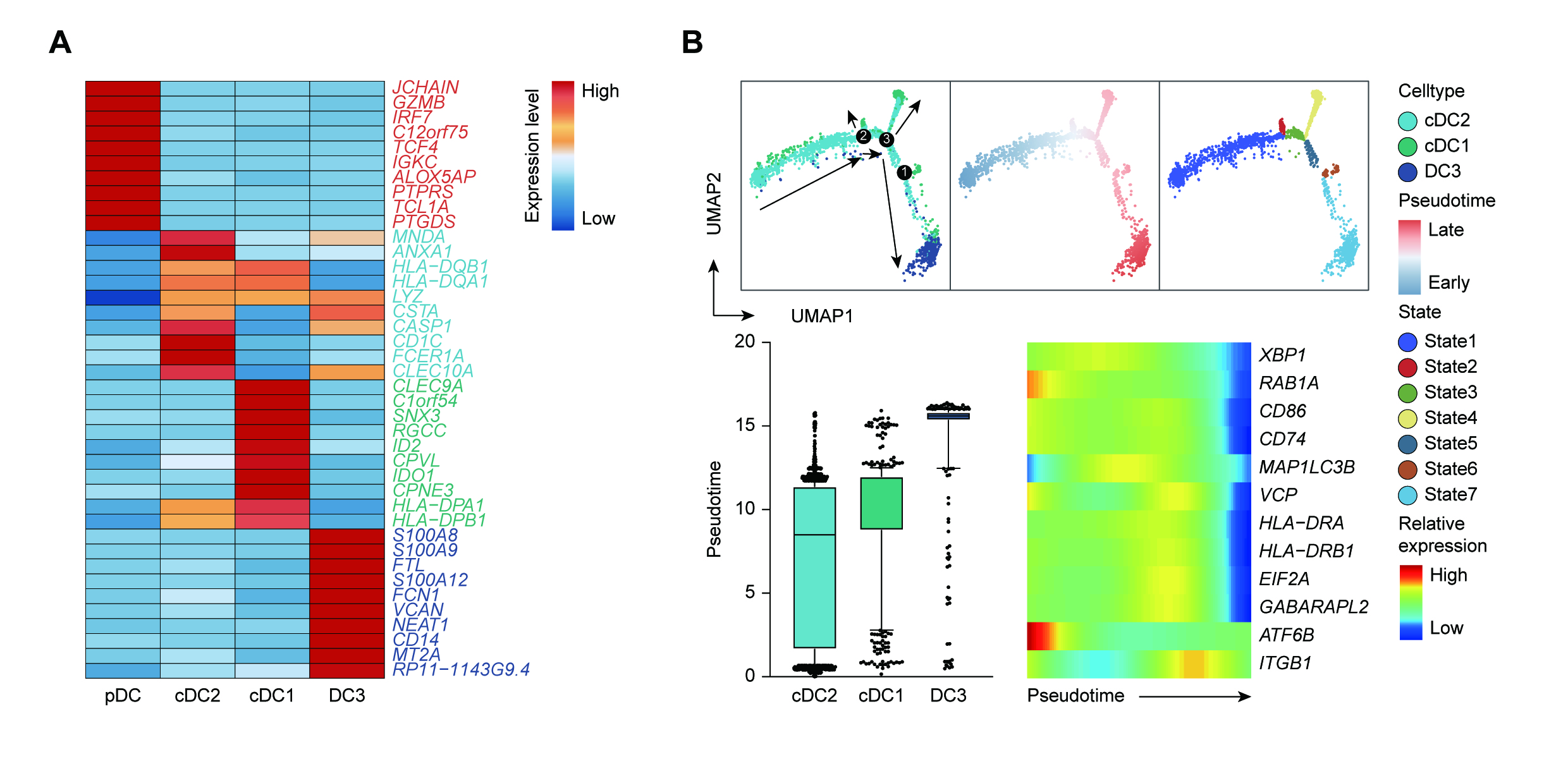


**Figure S18. ScRNA sequencing analyses of DC subtypes in human sepsis. A** Heatmap showing relative expression of DEGs among all DC subtypes (blue, low expression level; red, high expression level). **B** Developmental trajectory of color-coded DC subsets by cell state and pseudo-time, illustrating the putative trajectory for cell transition states of DCs and the proportion of each subtype (upper panel). Bar plot showing the relative distribution of cDC1, cDC2, and DC3 along with the pseudotime (lower left panel). Heatmap displaying dynamic transitions in expression level of genes related to reticulophagy and DC activation along with the pseudotime (lower right panel) (blue, low expression level; red, high expression level).


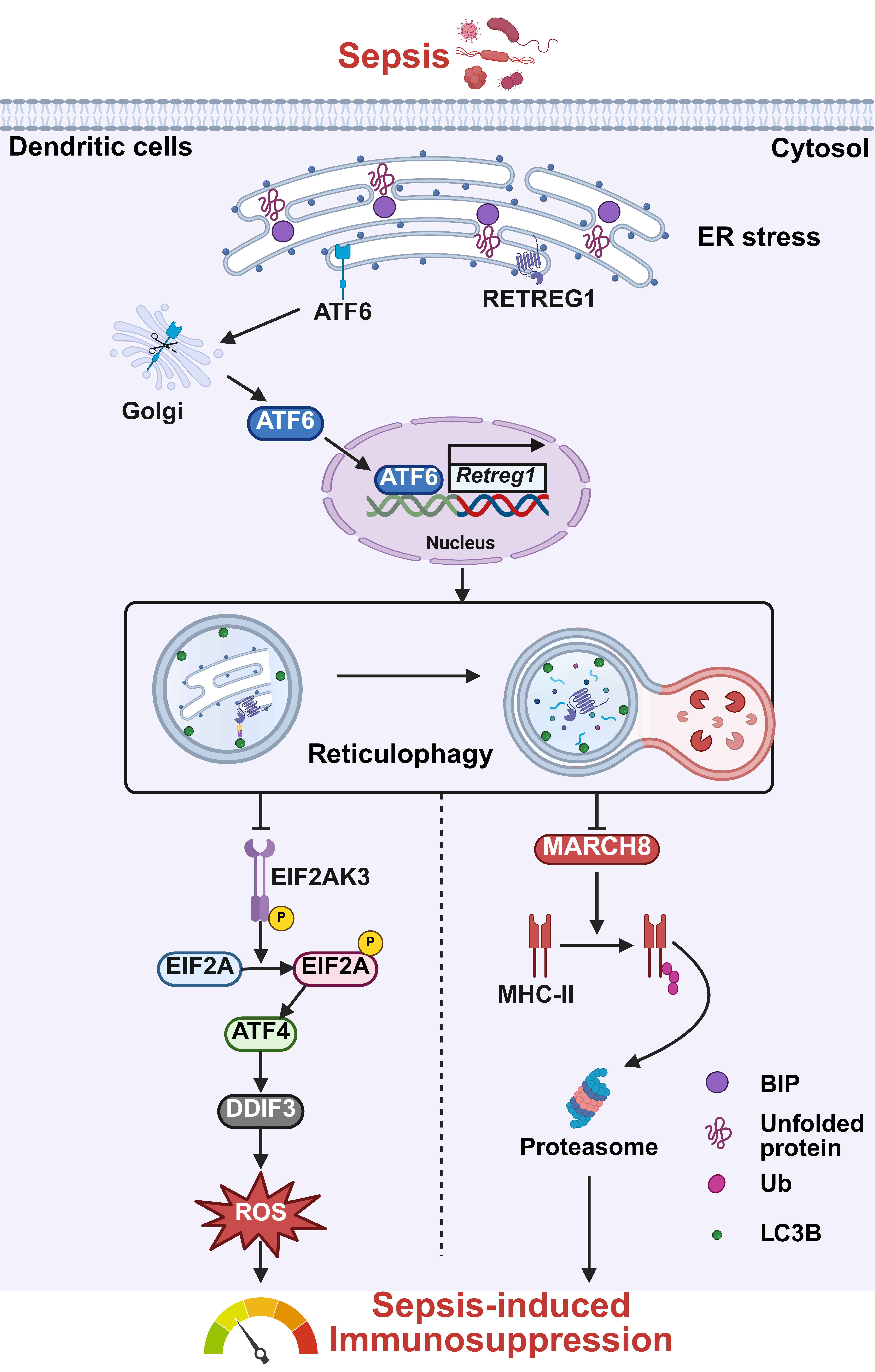


**Figure S19. Dysregulated RETREG1-mediated reticulophagy impairs immune function of DCs in sepsis.** ER stress is activated under septic exposure, initiating UPR pathways. Activated ATF6 migrates into the nucleus and binds to the promoter region of *Retreg1*, serving as a key regulator for modulating RETREG1 expression and reticulophagy. Consequently, RETREG1-mediated reticulophagy is mobilized to alleviate overactivated UPR through the EIF2AK3-ATF4-DDIT3-ROS axis and to modulate the ubiquitination of MHC-II in a MARCH8-dependent manner. Persistent septic challenge induces substantial impairment of RETREG1-mediated reticulophagy, which greatly compromises the functional status of DCs, thereby resulting in the development of sepsis-associated immunosuppression.
